# The mechanism for CO_2_ reduction over Fe-modified Cu(100) surfaces with thermodynamics and kinetics: a DFT study†

**DOI:** 10.1039/d0ra06319c

**Published:** 2020-09-01

**Authors:** Mei Qiu, Yi Li, Yongfan Zhang

**Affiliations:** Department of Chemistry, College of Science, Jiangxi Agricultural University Nanchang Jiangxi 330045 China qium@jxau.edu.cn; State Key Laboratory of Structural Chemistry, Fujian Institute of Research on the Structure of Matter, Chinese Academy of Sciences 350002 Fuzhou Fujian 350002 China; College of Chemistry, Fuzhou University Fuzhou Fujian 350116 China

## Abstract

The adsorption, activation and reduction of CO_2_ over Fe_*x*_/Cu(100) (*x* = 1–9) surfaces were examined by density functional theory. The most stable structure of CO_2_ adsorption on the Fe_*x*_/Cu(100) surface was realized. The electronic structure analysis showed that the doped Fe improved the adsorption, activation and reduction of CO_2_ on the pure Cu(100) surface. From the perspective of thermodynamics and kinetics, the Fe_4_/Cu(100) surface acted as a potential catalyst to decompose CO_2_ into CO with a barrier of 32.8 kJ mol^−1^. Meanwhile, the first principle molecular dynamics (FPMD) analysis indicated that the decomposition of the C–O1 bond of CO_2_ on the Fe_4_/Cu(100) surface was only observed from 350 K to 450 K under a CO_2_ partial pressure from 0 atm to 10 atm. Furthermore, the results of FPMD analysis revealed that CO_2_ would rather decompose than hydrogenate when CO_2_ and H co-adsorbed on the Fe_4_/Cu(100) surface.

## Abstract 2

The adsorption, activation and reduction of CO_2_ over Fe_*x*_/Cu(100) (*x* = 1–9) surfaces were examined by density functional theory.

## Graphical abstract

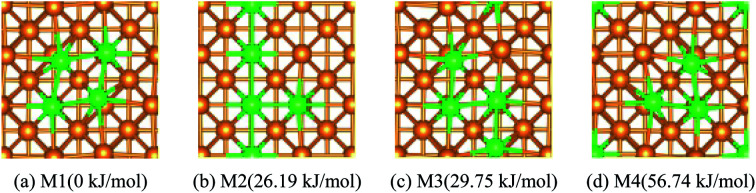

## 1. Introduction

Reducing the concentration of CO_2_ in the atmosphere has attracted significant attention, because as the major component of greenhouse gas, excessive emissions of CO_2_ have contributed significantly to environment degradation in the past decades, such as global warming, and melting of glaciers. The conversion of CO_2_ as a C resource to synthesize more valuable chemical raw materials not only solves the major crisis of global greenhouse gases and energy shortage, but also provides great opportunities and challenges for exploring novel catalysts and developing a modern catalytic industry.^1^

Because CO_2_ is a thermodynamically stable molecule, it is difficult to utilize CO_2_ as the C resource.^2^ Currently, five ways to reduce CO_2_ have been reported: (i) electrocatalytic reduction,^3–8^ (ii) photocatalytic reduction,^2,9–11^ (iii) thermal catalytic reduction,^12–15^ (iv) enzymatic reduction^16,17^ and (v) photoelectrocatalytic reduction.^18^ In these cases, the catalyst plays a major role in CO_2_ activity and reduction.

Numerous experiments and theories have been used to investigate the adsorption, activation and reduction of CO_2_ on transition metal–based catalysts.^19–27^ For instance, Fierro systematically investigated the adsorption of CO_2_ on the Co(100), Co(110) and Co(111) surfaces.^19^ The results indicated that the adsorption configuration of CO_2_ on the substrate was sensitive, especially the Co(110) surface. The experimental results of Rasmussen showed that the Cu(100) surface was able to decompose CO_2_ into CO and O_2_.^22^ Roberts demonstrated that CO_2_ could be decomposed into CO on the Ni(100) surface rather than on the Ni(111) surface.^23^ Ding asserted that a weak interaction was formed between CO_2_ and the Ni(110) surface.^24^ Glezakou investigated the mechanism of adsorption and activation of CO_2_ on the Fe fcc(100) surface.^25^ Their results indicated that CO_2_ on the Fe(100) surface was activated spontaneously. Wilson investigated the reduction of CO_2_ into CO on Co(100), Ni(100) and Cu(100) surfaces.^26^ The calculated results revealed an interesting trend between reaction energy and the total reaction barrier from Fe to Cu and that reactions tended to be less exergonic. Additionally, Co and Ni were more favorable to decompose CO_2_ into CO.

Except for the single metal catalysts for CO_2_, Great effort has been devoted to improve the catalytic performance of bimetallic catalysts for CO_2_ activation and catalysis.^27,28^ Nerlov pointed out that the performance of Cu–Ni bimetallic catalyst was more than 60 times greater than the pure Cu.^29–31^ Liu found that the introduction of Pd, Rh, Pt, and Ni metals on the Cu(111) surface promoted the methanol production.^32^ Our previous reports revealed that the introduction of a second metal could improve the interaction between CO_2_ and the Cu(100) surface.^33,34^ Additionally, the results also showed that the interaction between CO_2_ and the Co_*n*_/Cu(100) surface was structure sensitive for the reduction of CO_2_ molecules and the Co_4_/Cu(100) surface was the potential catalyst for the reduction of CO_2_. The results of Song demonstrated that the properties of CO_2_ conversion on Fe–Ni and Fe–Co catalysts were similar, and CO* and HCOO* were the preferred intermediates.^35,36^

On the basis of these reports, in this work the density functional theory calculations was employed to understand the activation and reduction of CO_2_ on the Cu(100) surface with embedded small Fe atoms. The result of a recent STM experiment showed that small Co clusters can be formed in the first layer of the Cu(100) surface through the vacancy-mediated diffusion of Co atoms.^37^ The similarity of the Co/Cu(100) and Fe/Cu(100) epitaxial systems suggests that the diffusion of embedded Fe atoms also leads to the formation of small nanostructures.^38^ Firstly, the most stable structure of Fe_*x*_/Cu(100) (*x* = 1–9) was generated using the first principle molecular dynamics (FPMD) method. Then the adsorption energetics and geometry, vibrational frequencies analysis, charge transfers and d-band center of CO_2_ over a series of Fe_*x*_/Cu bimetallic surfaces were analyzed. The activation energy barrier for the CO_2_ reduction and the Brønsted–Evans–Polanyi (BEP) relationships between the kinetic parameters for CO_2_ reduction over the Fe_*x*_/Cu(100) systems were investigated. And then the optimal temperature and partial pressure for CO_2_ reduction on the Fe_4_/Cu(100) surface was explored in detail. Lastly, the hydrogenation of CO_2_ and residual CO or O was considered in the paper.

## 2. Computation details

### 2.1. Method

All of the density functional theory (DFT) calculations using the Vienna *ab initio* simulation package (VASP)^39–41^ were according to projector-augmented wave DFT (PAW-DFT).^42–44^ The Perdew–Burke–Ernzerhof (PBE) functional based on the generalized gradient approximation was employed.^45^ The kinetic energy cutoff of 400 eV for the plane-wave expansion was set. In all systems, the effects of dipole correction, spin polarization in the surface normal direction and the van der Waals correction were considered by using DFT-D2 method.^46,47^ Geometrical structure was optimized until the energy change and the maximum force were less than 10^−6^ eV and 0.02 eV Å^−1^, respectively. The 5 × 5 × 1 *k*-points with Monkhorst–Pack method were used.^48^

### 2.2. Surface model

Previous studies reveal that the Cu(100) surface is the most potential value to be reduction of CO_2_ molecule among Cu(100), Cu(110) and Cu(111) surfaces.^34^ Thus, the Cu(100) surface with a *p*(3 × 3) supercell and five atomic layers was employed. For the Fe_*x*_/Cu(100) (*x* = 1–9) surfaces, Cu atoms on top-layer of the pure Cu(100) surface were substituted by several Fe atoms. In the geometry optimizations, the top three layers were completely relaxed in all directions and the bottom two layers were fixed in their bulk position. The vacuum of 15 Å between the adjacent slabs was set along the *c*-axis direction to avoid periodic interactions.

The adsorption energy (*E*_ads_) for the CO_2_ molecule on the Fe_*x*_/Cu(100) surface was defined eqn (1), as follows:

1 *E*_ads_ = *E*_CO_2__ + *E*_Slab_ − *E*_CO_2_/Slab_

where, *E*_CO_2__, *E*_Slab_ and *E*_CO_2_/Slab_ represent the total energy of the free CO_2_ molecule in the gas phase, the clean surface and the slab with adsorbed CO_2_, respectively. Therefore, a negative value of *E*_ads_ indicates an endothermic adsorption and a positive value refers to an exothermic adsorption.

The reduction of CO_2_ on the Fe_*x*_/Cu(100) surface was involved in the formations of CO and HCOO, namely, 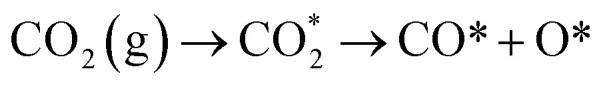 and 
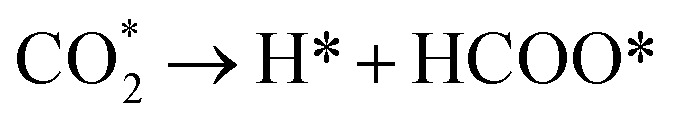
 (where X* represents X species adsorbed on substrate). The corresponding activation energy barrier was calculated from the relative energy of the transition state (TS) with respect to the sum of the energies of the initial structure (IS) (CO_2_ adsorbed on the Fe_*x*_/Cu(100) surface), namely,

2 *E*_a_ = *E*_TS_ − *E*_IS_

All the transition states were determined using the climbing image nudged elastic band (CI-NEB)^49,50^ and DIMER^51,52^ methods and performed a vibrational frequency analysis to confirm that the predicted transition state to the first-order saddle point in the reaction path. Additionally, the Bader charge analysis using the code developed by Henkelman and co-workers was employed to quantify the charge transfer between the substrates and CO_2_ molecule.^53–55^

### 2.3. Microkinetic model

The rate constants for CO_2_ dissociation using the harmonic transition state theory^56–58^ was analyzed as shown in eqn (3):3
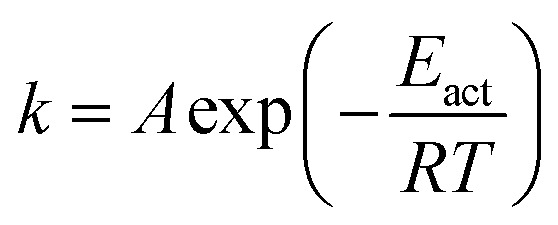
where, *A* represents the pre-exponential factor. According to the harmonic transition state theory, the pre-exponential factor (*A*) can be estimated using the following formula:4
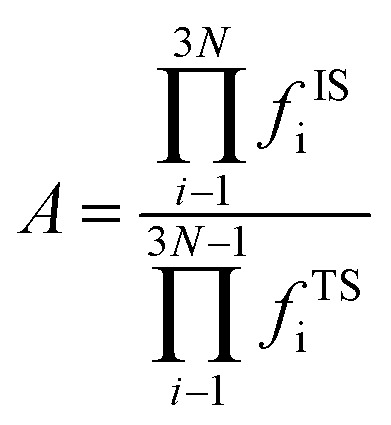
where *f*^IS^_i_ and *f*^TS^_i_ were the vibrational frequencies at the IS and the TS. Note that the imaginary frequency in TS was excluded.

The actual activation barrier (*E*_act_), including the entropy (ΔTS), the zero point energy (ZPE) and enthalpy 
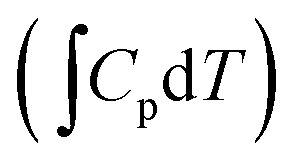
 corrections, was calculated as follows:5
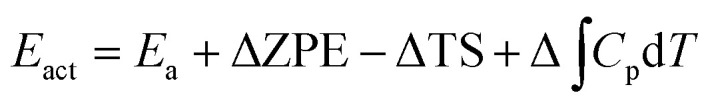
where, *S* and *C*_p_ represent the entropy and the heat capacity, respectively. The zero point energy, entropy and enthalpy correction are calculated as follows,^57^ respectively:6
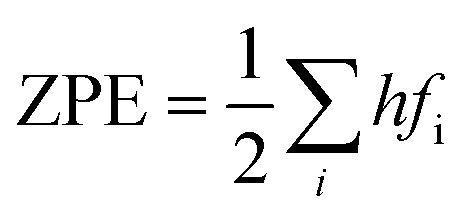
7
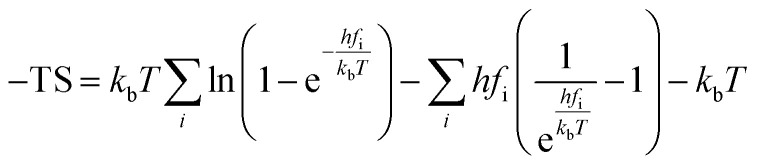
8
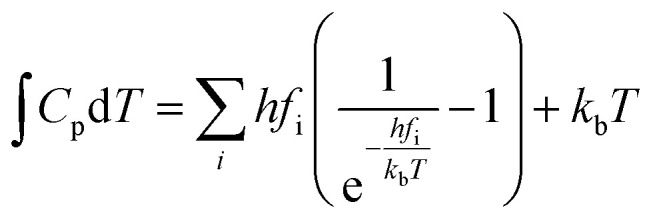
where *f*_i_ was the vibrational frequency and i represents the different modes of vibration.

To calculate the relative concentrate (*θ*_CO_) of CO on the Fe–Cu bimetallic system, the steady state approximation was adopted in this work.^59^ The total amount of metal catalytic sites in the reaction was considered as a constant and the sum of the occupied (*θ*_*x*_) and the free metal (θ_*_) sites were defined as following eqn (9):^60^

9 *θ*_CO_2__ + 2*θ*_CO_ + θ_*_ = 1

where *θ*_CO_2__ was obtained by *θ*_CO_2__ × *K*_CO_2__ × *P*_CO_2__ × θ_*_ and 
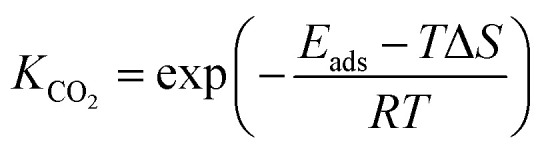
.^61^ The partial pressure (*P*_CO_2__) of CO_2_ from 0 to 10 atm was set.

For the elementary reaction step 
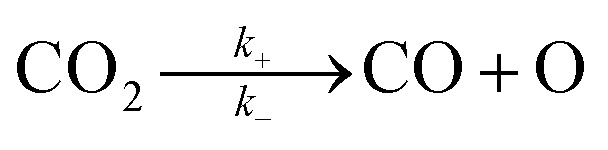
 (rate constants, 
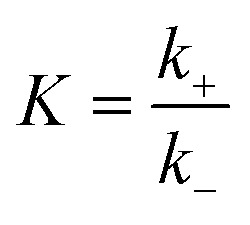
), according to the steady-state approximation, the equation was as follows:10
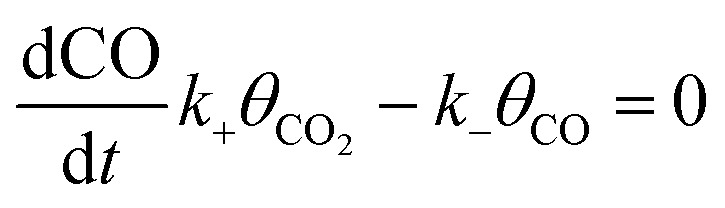


Thus,11
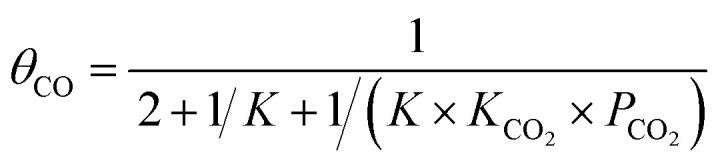


## 3. Results and discussion

The most stable structure of the different coverage (*n*) for Fe dopant on the top-layer of the Cu(100) surface was discussed briefly.

**Fig. 1:**
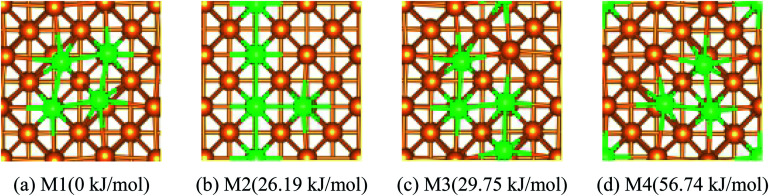
Top views and the relative energies for four different arrangements of four Fe atoms embedded in the toplayer of Cu(100) surface, Cu Orange Fe green.

If *n* > 1/9 ML, more than one possible structure of Fe could be doped in the Cu(100) surface. Optimal doping structure for the different coverage of Fe dopant was predicted after extensive study of the various arrangements of Fe atoms embedded in the top-layer of the Cu(100) surface. Taking Fe_4_/Cu(100) as an example, four possible configurations for the Fe_4_/Cu(100) surface, namely: M1 ∼ M4, (see Fig. 1), were considered. In the M1 model, four Fe atoms tended to gather together and form a square nanocluster. For the M2 and M3 structure, the four Fe atoms exhibited the T- and Z-type structure, respectively. In the M4 structure, it was seen as the Fe_3_ trimer and an isolated Fe atom. The calculated relative energies (Fig. 1) indicated that the energetically most favorable configuration among the M1 ∼ M4 models was the M1. The most stable structure for the Fe_*x*_/Cu(100) surfaces was determined by the similar approach and shown in Fig. S1.† Interestingly, from the perspective of thermodynamic, Fe dopant tended to arrange together when the *n* values increased from 1/9 to 1 ML. Therefore, we focused on the adsorption, activation and reduction of CO_2_ molecule on the most stable configuration for the Fe_*x*_/Cu(100) surfaces.

### 3.1. Adsorption configurations of CO_2_ on Fe_*x*_/Cu(100) surfaces

**Fig. 2:**
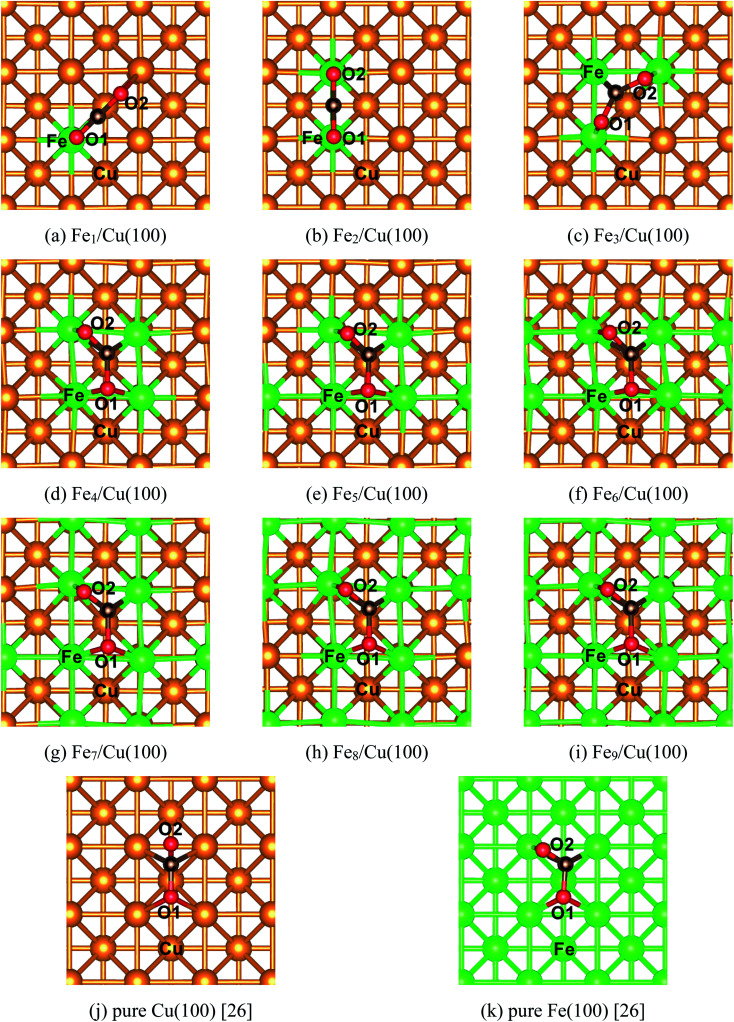
Top views of the most stable configurations of CO_2_ adsorbed on the different Fe_*x*_Cu(100) surfaces, as well as on the pure Cu(100) and Fe(100) surfaces with fcc structure.

The most stable structure of CO_2_ on the Fe_*x*_/Cu(100) (*x* = 1–9) surface was presented in Fig. 2, including CO_2_ on the pure Cu(100) and Fe fcc(100) surfaces. It was clearly observed in Fig. 2 that the adsorption behavior of CO_2_ on the Fe_*x*_/Cu(100) surface was sensitive to the coverage of Fe atoms when the coverage of Fe atoms was less than 4/9 ML (Fig. 2(a)–(c)). While the coverage was more than 4/9 ML (Fig. 2(d)), the adsorption configuration of CO_2_ on the Fe_*x*_/Cu(100) surface (Fig. 2(e)–(i)) was similar to the structure of CO_2_ on the Fe_4_/Cu(100) surface. Herein, it was worth noting that the O atoms in the CO_2_ molecule tended to bind with the Fe atoms on the substrate *via* forming Fe–O adsorption bonds because the strength of the Fe–O bond was stronger than the Cu–O bond (*e.g.*, in a diatomic molecule, the dissociation energies of the Fe–O bond and Cu–O bond were about 390.4 and 269.0 kJ mol^−1^, respectively), indicating that the introduced Fe atoms were the major center of CO_2_ adsorption and activation.

After CO_2_ adsorption, some electrons were transferred from the substrate to CO_2_ moiety, leading to the bent structure with the O–C–O bond angle from 118° to 135° (Table 1). Furthermore, the C–O1 bond and the C–O2 bond was stretched to about 1.253 to 1.364 Å and 1.253 to 1.300 Å, respectively. Especially the C–O1 bond, its distance was gradually elongated by 0.2 Å compared with the distance of 1.176 Å in gas-phase CO_2_ molecule when the coverage of the Fe atoms was more than 3/9 ML, which meant that the C–O1 bond was activated after CO_2_ adsorption. Meanwhile, it was also noticed that when the coverage of the Fe atoms was more than 3/9 ML, the extra interaction between the O2 atom in the CO_2_ moiety and the Fe atom was established and the corresponding Fe–O2 bond length was 2.014 Å (Fe_4_/Cu(100)), 2.000 Å (Fe_5_/Cu(100)), 1.987 Å (Fe_6_/Cu(100)), 1.974 Å (Fe_7_/Cu(100)), 1.961 Å (Fe_8_/Cu(100)) and 1.965 Å (Fe_9_/Cu(100)). Among these Fe–O2 bonds, the distance of the Fe–O2 bond for CO_2_ on the Fe_4_/Cu(100) was the longest, requiring the less energy barrier to decompose CO_2_. The calculated adsorption energy (see Table 1) increased monotonously as an increase of the coverage for Fe atoms, suggesting that introducing Fe atoms in the pure Cu(100) surface could improve the bonding strength of CO_2_ to the substrate. Compared with the adsorption energy of CO_2_ on the pure Cu(100) (*E*_ads_ = −72.4 kJ mol^−1^) and Fe fcc(100) (*E*_ads_ = 139.8 kJ mol^−1^) surfaces,^26^ the binding strength of CO_2_ on the substrate was enhanced while the coverage of Fe atoms was more than 3/9 ML.

**Table 1:** Some optimized bond lengths (Å), O–C–O bond angle (degrees) and the calculated adsorption energies (*E*_ads_, in kJ mol^−1^) of CO_2_ molecules on pure Fe(100), Cu(100) and different Fe_*x*_/Cu(100) bimetallic surfaces

System	*d*_C–O1_ a	*d*_C–O2_	∠O–C–O	*d*_Fe–C_	*d*_Fe–O1_	*d*_Cu–O2_	*d*_Fe–O2_	*E*_ads_
Fe_1_/Cu(100)	1.258	1.271	134.5	1.929	2.015	2.163	—	84.5
Fe_2_/Cu(100)	1.253	1.253	138.9	2.041 (×2)^b^	2.091	—	2.092	115.9
Fe_3_/Cu(100)	1.296	1.295	124.4	1.939	1.969	—	1.971	135.3
Fe_4_/Cu(100)	1.356	1.293	121.3	2.113/1.936	2.039/2.040	—	2.014	145.3
Fe_5_/Cu(100)	1.354	1.296	121.2	2.116/1.936	2.046/2.048	—	2.000	148.6
Fe_6_/Cu(100)	1.351	1.299	121.2	2.160/2.050	2.048/2.050	—	1.987	153.0
Fe_7_/Cu(100)	1.362	1.296	118.5	2.115/1.938	2.047/2.013	—	1.974	147.4
Fe_8_/Cu(100)	1.361	1.300	118.3	2.167/1.924	2.028/2.009	—	1.961	154.8
Fe_9_/Cu(100)	1.364	1.299	118.3	2.138/1.924	2.038/2.009	—	1.965	150.8
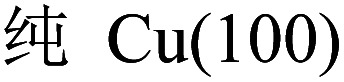 c	1.322	1.220	128.4	2.157	2.098	—	—	−72.4
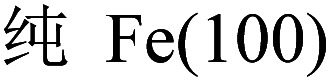 c	1.340	1.290	120.5	2.190/1.950	2.040/2.080	—	1.990	139.8

a The symbols O1 and O2 represent the two oxygen atoms of the CO_2_ moiety (see Fig. 2). For the free CO_2_ molecule, the optimized length of the C–O bond is 1.176 Å.

b The number of bonds is shown in parentheses.

c The data origin from the ref. 20.

### 3.2. Electronic structures of CO_2_ on Fe_*x*_/Cu(100) surfaces

#### 3.2.1. D-Band center analysis

**Fig. 3:**
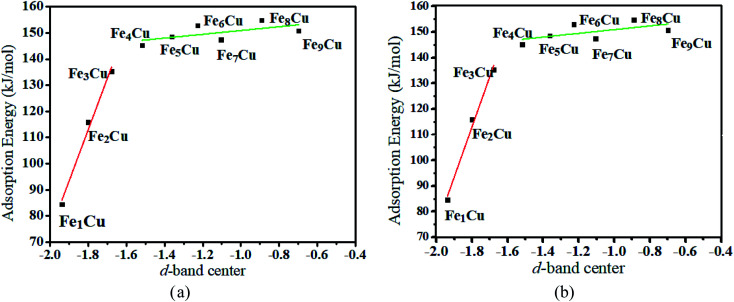
Relationship between (a) the total d-band center (b) the d-band center for beta-orbitals of different Fe_*x*_/Cu(100) surface and the adsorption energies of CO_2_ molecule.

**Fig. 4:**
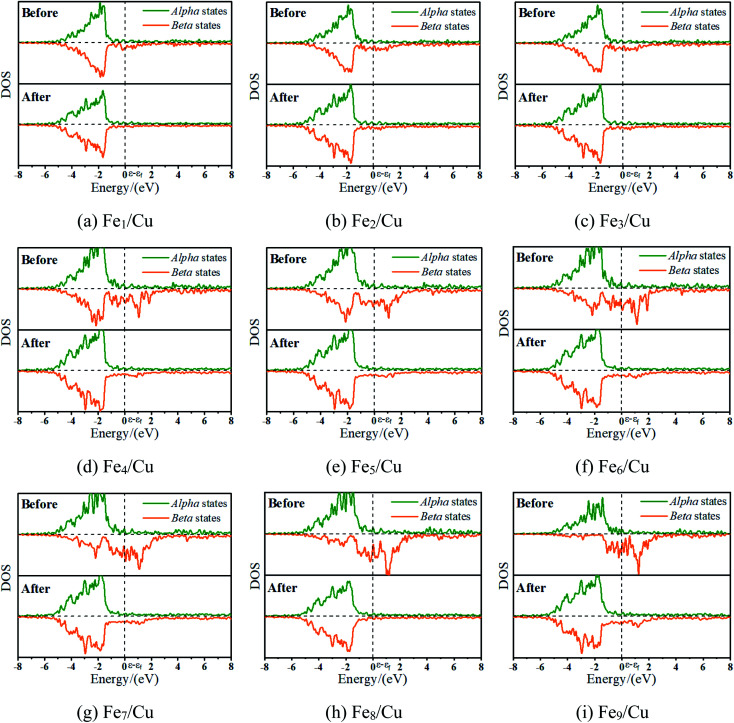
The density of states for the toplayer metallic atoms in clean Fe_*x*_/Cu(100) and CO_2_/Fe_*x*_/Cu(100) surfaces.

To interpret a variation in adsorption energy as an increase of coverage for Fe atoms, the position of the d-band center of the Fe_*x*_/Cu(100) surface was calculated. As well-known, the d-band center model was used widely to understand the bond formation on a transition metal surface, this is, the higher the d-band center, the stronger the adsorption bond.^33^ As shown in Fig. 3(a), the value of the d-band center for the Fe_*x*_/Cu(100) surface was in the range of 2.0 to −0.7 eV and tended to increase with an increase of Fe atoms on the Cu(100) surface. Although the coverage exceeded 3/9 ML, the variation of adsorption energy was small as the increase of position of the d-band center because of the same structure of the CO_2_ on Fe_*x*_/Cu(100) surface. Furthermore, to gain a deeper understanding of the active state of the Fe_*x*_/Cu(100) surface, the position of the d-band center for alpha (α) and beta (β) states in 3d-orbitals was extracted from the total position of the d-band center. As shown in Fig. S2† and 3(b), the trend of the variation of the d-band center for β-states orbitals was consistent with that of the total d-band center, which meant that CO_2_ was adsorbed on the Fe_*x*_/Cu(100) surface by interaction with the β-states orbitals of the doped Fe atoms. Furthermore, the calculated density of states (DOS) in Fig. 4 confirmed that, after CO_2_ adsorption on Fe_*x*_/Cu(100) surface, CO_2_ tended to interact with the β-states of the substrates.

#### 3.2.2. Bader charge analysis

**Fig. 5:**
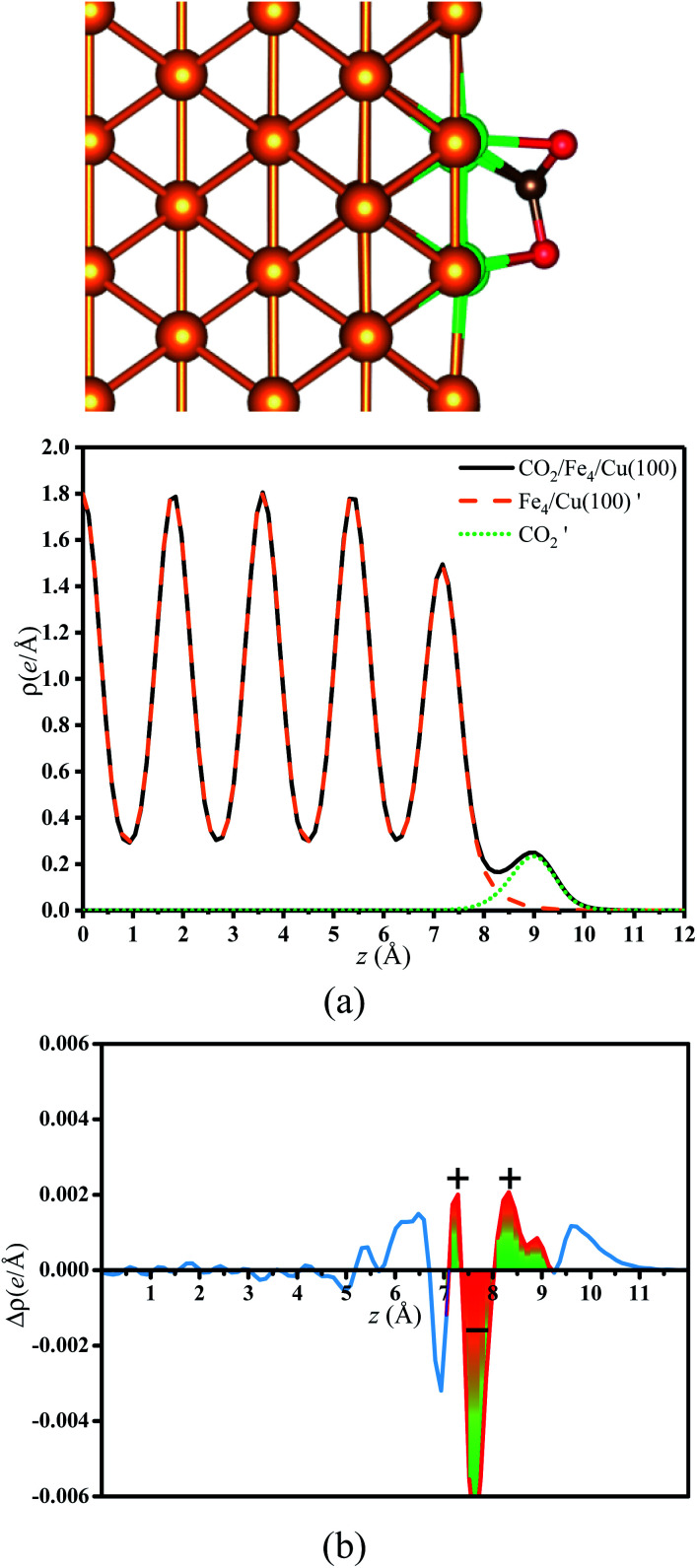
(a) Charge density curves of isolated Fe_4_/Cu(10)′ surface, 
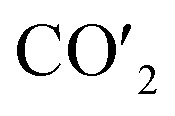
 moiety and CO_2_/Fe_4_/Cu(100) surface. (b) Charge density difference curve of CO_2_/Fe_4_/Cu(100) surface: 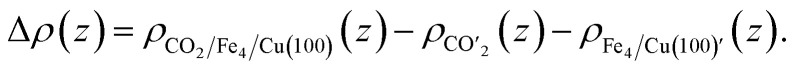 Fe_4_/Cu(100) surface denotes from the CO_2_/Fe_4_/Cu(100) system and 
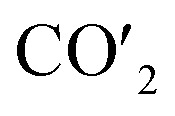
 is the bent CO_2_ moiety of the same CO_2_/Fe_4_/Cu(100) system.

As mentioned, the CO_2_ on the substrate exhibited the bent structure, indicating that some electrons were transferred to CO_2_ from substrate. To verify this conclusion more clearly, the charge density of CO_2_ on the Fe_*x*_/Cu(100) surface was calculated and shown in Fig. S3–S10.† Taking CO_2_ on the Fe_4_/Cu(100) surface as an example, Fig. 5(a) represents the charge density map of CO_2_ adsorbed on the Fe_4_/Cu(100) surface (the *x*-axis represents the distance from the bottom to the top of the Fe_4_/Cu(100) system). Five high-density peaks in Fig. 5(a) were observed and each peak represented the charge density of one atomic layer in the Fe_4_/Cu(100) surface. Furthermore, a relatively small density peak is observed at ∼9.0 Å along the *x*-axis, which represented the charge density of the CO_2_ moiety. It was noted that an obvious interaction between CO_2_ and the Fe_4_/Cu(100) surface was observed at *z* = 7–9 Å. The charge density difference map (Δ*ρ*) in Fig. 5(b) changed from lightly positive to negative upon crossing the Fe_4_/Cu(100) interface (*z* = 7.0–8.0 Å) and reached a minimum at *z* = ∼8.6 Å. Then it changed from negative to positive in the region (*z* = 8–9 Å). This evolution indicated that the electrons flowed from the Fe_4_/Cu(100) surface to the CO_2_ moiety, forming the CO_2_^−^ anions.

To quantify these charges transferred, the Bader charge analysis for all systems was calculated, as shown in Table 2. As expected, CO_2_ obtained some electrons from the Fe_*x*_/Cu(100) surfaces. In general, as the coverage increased for Fe atoms, the electrons obtained by CO_2_ from the surface also increased. In particular, for the Fe_9_/Cu(100) surface, the transferred electrons reached the maximum (1.17*e*). The atomic charges for C and O atoms of CO_2_ moiety were also included in Table 2. It was obviously evident in Table 2 that the transferred electrons were significantly concentrated on C atom.

**Table 2:** Bader charges (*e*) of the CO_2_ moiety, the carbon and oxygen atoms of different Fe_*x*_/Cu(100) surfaces and the free CO_2_ molecule

	C	O1	O2	CO_2_ moiety
CO_2_	1.94	−0.97	−0.97	0
Fe_1_/Cu(100)	1.29	−1.07	−0.99	−0.77
Fe_2_/Cu(100)	1.24	−1.01	−1.01	−0.78
Fe_3_/Cu(100)	1.14	−1.03	−1.04	−0.93
Fe_4_/Cu(100)	0.98	−1.06	−1.04	−1.12
Fe_5_/Cu(100)	0.90	−1.02	−1.00	−1.13
Fe_6_/Cu(100)	0.96	−1.08	−1.02	−1.14
Fe_7_/Cu(100)	0.92	−1.03	−1.04	−1.15
Fe_8_/Cu(100)	0.94	−1.08	−1.02	−1.16
Fe_9_/Cu(100)	0.94	−1.08	−1.03	−1.17
Pure Cu(100)^a^	1.47	−1.10	−1.00	−0.63
Pure Fe fcc(100)^a^	1.02	−1.12	−1.05	−1.16

a The data origin from ref. 20.

**Fig. 6:**
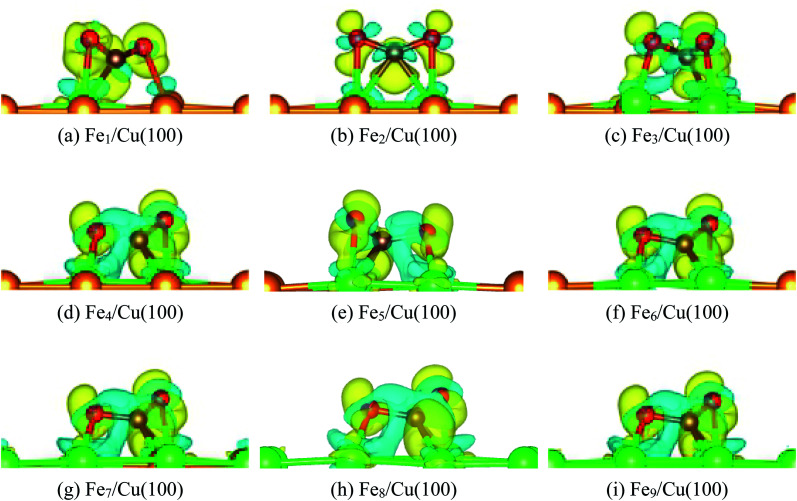
Charge density difference of CO_2_ on Fe_*x*_/Cu(100) (*x* = 1–9) surface.

Fig. 6 displays the charge density difference for CO_2_ moiety on Fe_*x*_/Cu(100) surface. If the coverage was less than 4/9 ML, the π-bond between C and O1 atoms was weakened slightly, especially the Fe_1_/Cu(100). Although the coverage was more than 3/9 ML, Fig. 6(d)–(i) clearly showed that the degree of activation for the π-bond was quite similar and was more larger than CO_2_ on the Fe_1∼3_/Cu(100) surface.

#### 3.2.3. Vibrational frequencies analysis

According to the Bader charge analysis, after CO_2_ adsorbed on the Fe_*x*_/Cu(100) surface, some electrons were transferred from the substrate to CO_2_ moiety and concentrated on the C atom, which weakened the C–O bond and further caused a red shift of the vibrational frequency for C–O bond, especially the C–O1 bond. Table 3 present the stretching vibration frequency of C–O1 and C–O2 bonds in CO_2_ moiety on each of the Fe_*x*_/Cu(100) surface. Compared with stretching vibration frequency of a free CO_2_ molecule, the stretching vibration frequency of the C–O1 and C–O2 bonds was red-shifted when the CO_2_ was adsorbed on the Fe_*x*_/Cu(100) surface. However, the variation of the stretching vibration frequency for C–O2 bond decreased lightly when the coverage was more than 3/9 ML. Compared with the stretching vibration frequency (1333 cm^−1^) of the gas CO_2_ molecule, the reducing value of the stretching vibration frequency for the C–O1 bond was 211 cm^−1^ (Fe_1_/Cu(100)), 181 cm^−1^ (Fe_2_/Cu(100)), 216 cm^−1^ (Fe_3_/Cu(100)), 316 cm^−1^ (Fe_4_/Cu(100)), 314 cm^−1^ (Fe_5_/Cu(100)), 308 cm^−1^ (Fe_6_/Cu(100)), 344 cm^−1^ (Fe_7_/Cu(100)), 340 cm^−1^ (Fe_8_/Cu(100)) and 354 cm^−1^ (Fe_9_/Cu(100)), respectively. Among these frequencies, the variation of the stretching vibration frequency of the C–O1 bond adsorbed on the Fe_9_/Cu(100) surface reached its maximal value, which was consistent with the variation in bond length and the number of transferred charges after adsorption. Thus, the more electrons were transferred to the CO_2_ molecule, the longer the C–O1 bond length was and the easier the stretching vibration frequency for the C–O1 bond was to redshift.

**Table 3:** The stretching vibration of the C–O1 and C–O2 bonds in CO_2_ moiety on Fe_*x*_/Cu(100) surface and free CO_2_

System	*v*_C–O1_/cm^−1^	*v*_C–O2_/cm^−1^
Free CO_2_^a^	1333	1333
Fe_1_/Cu(100)	1122	1610
Fe_2_/Cu(100)	1152	1152
Fe_3_/Cu(100)	1117	1117
Fe_4_/Cu(100)	1017	1305
Fe_5_/Cu(100)	1019	1296
Fe_6_/Cu(100)	1025	1283
Fe_7_/Cu(100)	989	1279
Fe_8_/Cu(100)	993	1258
Fe_9_/Cu(100)	979	1261

a The data origin from NIST database: https://webbook.nist.gov/chemistry.

### 3.3. Decomposition of CO_2_ on Fe_*x*_/Cu(100) surfaces

#### 3.3.1. Transition state analysis

**Fig. 7:**
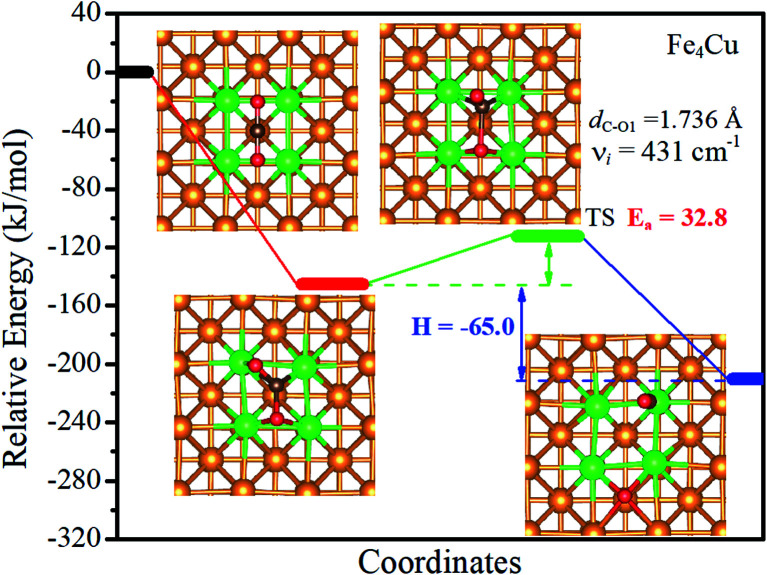
Calculated reaction paths of CO_2_ dissociation on the Fe_4_/Cu(100) surface. The zero of energy is set to the total energy of the isolated surface and CO_2_ molecule in the gas phase.

The minimum potential energy map for the decomposition of CO_2_ molecules on the Fe_*x*_/Cu(100) surfaces was determined using the CI-NEB and DIMER methods and shown in Fig. 7 and S11† According to the Fe_1_/Cu(100) surface shown in Fig. S11(a),† the O atom bonded to three Cu atoms and one Fe atom (4-fold site), whereas the CO moiety was adsorbed between two adjacent Cu atoms. On all other Fe_*x*_/Cu(100) surfaces, the O atom was still bonded to four metal atoms to form the 4-fold site and the C atom of CO moiety was co-adsorbed to the substrate with an incline through the Fe–C adsorption bond. The reaction energy (H) of CO_2_ decomposition on the Fe_*x*_/Cu(100) surface was calculated, as shown in Fig. 7 and S11.† For the Fe_1_/Cu(100) surface, the highest reaction energy was −4.9 kJ mol^−1^, followed by the Fe_3_/Cu(100) surface (−36.1 kJ mol^−1^) and the Fe_2_/Cu(100) surface (−43.1 kJ mol^−1^). When the coverage of Fe atoms was more than 3/9 ML, the reaction energy could be significantly increased with −65.0 kJ mol^−1^ for the Fe_4_/Cu(100), −62.3 kJ mol^−1^ for the Fe_5_/Cu(100) surface, −70.7 kJ mol^−1^ for the Fe_6_/Cu(100) surface, −121.0 kJ mol^−1^ for the Fe_7_/Cu(100) surface, −122.5 kJ mol^−1^ for the Fe_8_/Cu(100) surface and −127.6 kJ mol^−1^ for the Fe_9_/Cu(100) surface. It was noteworthy that the reaction energy of the Fe_*x*_/Cu(100) surface increased with the coverage of the Fe atom increased on the Cu(100) surface, indicating that the introduced Fe atom improved the reaction energy of CO_2_ decomposition. It may be reasonable that, as the coverage of Fe atoms increased, the number of Fe–O bonds increased and the strength of Fe–O bond was stronger than that of Cu–O bond, thereby reaction energy was increased for CO_2_ decomposition from Fe_1_/Cu(100) to Fe_9_/Cu(100) surfaces. From the thermodynamic point of view, the negative reaction energy indicated that the Fe_*x*_/Cu(100) surface would be favorable to decompose CO_2_ into CO, especially the Fe_9_/Cu(100) surface.

The activation energy barrier (*E*_a_) of CO_2_ decomposition on the Fe_*x*_/Cu(100) surface was determined and shown in Fig. 7 and S11.† In addition, the vibration frequencies of all transition states were calculated to ensure that the predicted TS corresponds to the first-order saddle point in the reaction path and the imaginary frequency (*ν*_i_) was also shown in Fig. 7 and S11.† For the Fe_1_/Cu(100) surface, only one TS for the C–O1 bond decomposition was discovered. The activation energy barrier (relative to the energy of the CO_2_ adsorption) of CO_2_ decomposed on the Fe_1_/Cu(100) surface was 45.6 kJ mol^−1^. For the Fe_2_/Cu(100) and Fe_3_/Cu(100) surfaces, the activation energy barrier increased to 59.9 kJ mol^−1^ and 63.7 kJ mol^−1^ compared with the Fe_1_/Cu(100) surface, respectively. When the coverage reached 4/9 ML, the activation energy barrier reached the minimum value (32.8 kJ mol^−1^). The transition state of CO_2_ decomposition on the Fe_*x*_/Cu(100) surface was extremely similar to the CO_2_ on the Fe_4_/Cu(100) surface when the coverage was greater than 4/9 ML. The corresponding activation energy barrier from the Fe_5_/Cu(100) to Fe_9_/Cu(100) systems was 35.4 kJ mol^−1^, 39.7 kJ mol^−1^, 34.7 kJ mol^−1^, 40.2 kJ mol^−1^ and 35.0 kJ mol^−1^, respectively. The results showed that the activation energy barrier of all systems exhibited an inverted “S” shape, which was consistent with the variation in the distance between Fe and O2 atoms, that is, the shorter the distance of the Fe–O2 adsorption bond, the larger the activation energy barrier. For instance, the length (2.014 Å) of the Fe–O2 adsorption bond formed on the Fe_4_/Cu(100) surface was the largest among the Fe_*x*_/Cu(100) system, which indicated that the O2 atom may need to consume less additional energy to “escape” out of the substrate. Therefore the activation energy barrier of CO_2_ decomposition on the Fe_4_/Cu(100) surface was minimal. In addition, Fe dopant introduced on the Cu(100) surface significantly reduced the activation energy barrier of CO_2_ decomposition compared with the barrier of CO_2_ on the pure Cu(100) surface with the barrier of 92.9 kJ mol^−1^ and the Fe(100) surface with the barrier of 113.4 kJ mol^−1^, especially the Fe_4_/Cu(100) system.^26^ Compared to CO_2_ on the Co_4_/Cu(100) surface with the barrier of 18.7 kJ mol^−1^,^33^ the activation energy barrier for CO_2_ on the Fe_*x*_/Cu(100) is more than 14.1 kJ mol^−1^. The main reason is that bond strength of Fe–O (in diatomic molecules: 390.4 kJ mol^−1^) is stronger than that of Co–O (in diatomic molecules: 384.5 kJ mol^−1^), which led to be the extra barrier formed by Fe–O bond to overcome.

**Fig. 8:**
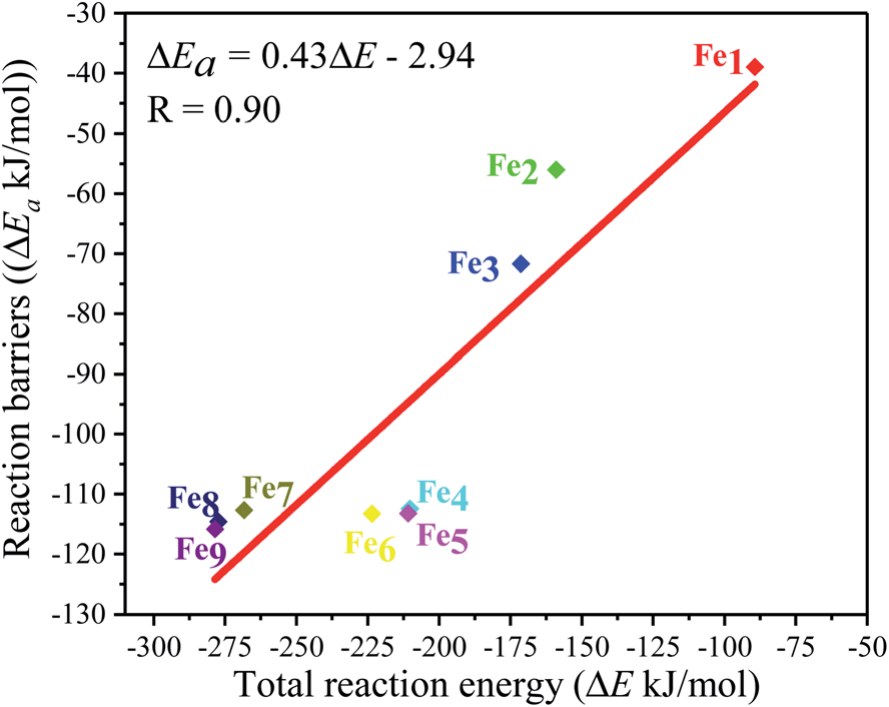
Brønsted–Evans–Polanyi (BEP) relationship for the C–O1 bond scission of CO_2_ on the Fe_*x*_/Cu(100) (*x* = 1–9) surfaces.

Moreover, the reaction energy barrier (Δ*E*_a_ = *E*_TS_ − *E*_Slab_ − *E*_CO_2__) and the total reaction energy (Δ*E* = *E*_ads_ + *H*) followed a linear relationship for the C–O bond scission and the BEP relationship was established: Δ*E*_a_ = 0.4Δ*E* − 2.5 (kJ mol^−1^) (see Fig. 8). This BEP relationship played an important role in estimating the reaction barrier of the C–O bond scission on other metal surfaces. Furthermore, the results in Fig. 3 showed an increasing trend as the number of Fe-dopants increased. It was worth noting that although the Fe_4_/Cu(100) surface did not have the lowest reaction energy barrier and total reaction energy, the Fe_4_/Cu(100) surface had the lowest activation energy barrier for CO_2_ decomposition, which facilitated the decomposition of CO_2_.

#### 3.3.2. Micro kinetics analysis

To further explore the mechanism of CO_2_ decomposition on the Fe_*x*_/Cu(100) surfaces from the perspective of dynamics, a microkinetic analysis based on the DFT studies was employed on the most favorable path of CO_2_ decomposition.^62,63^ In the microkinetic model, the temperatures ranging from 250 to 1000 K was adopted to investigate its impact.

**Fig. 9:**
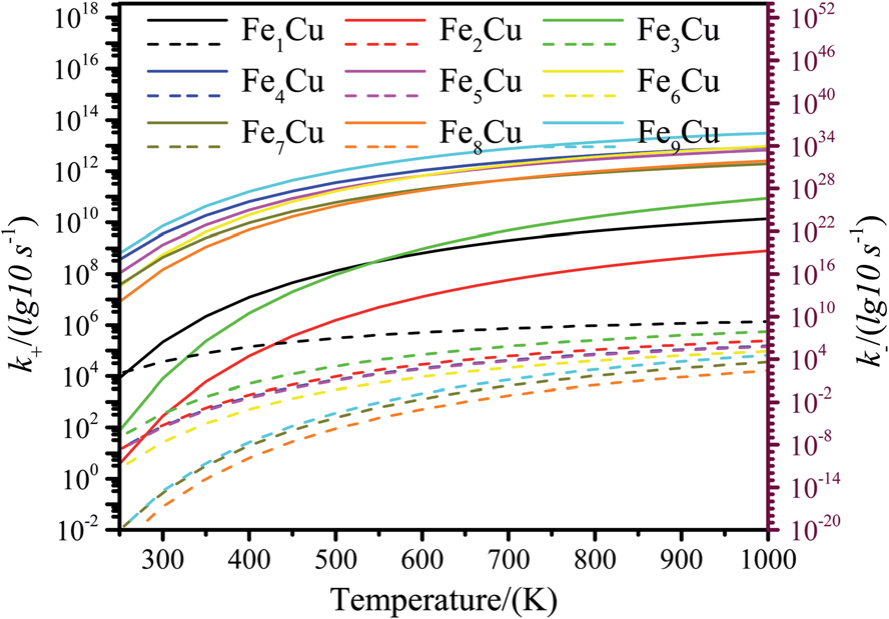
The forward and reverse rate constants for CO_2_ decomposition on the Fe_*x*_/Cu(100) surfaces at temperature from 250 K to 1000 K. (The solid line represents the positive equilibrium constants and the dashed line is the inverse equilibrium constants).

**Fig. 10:**
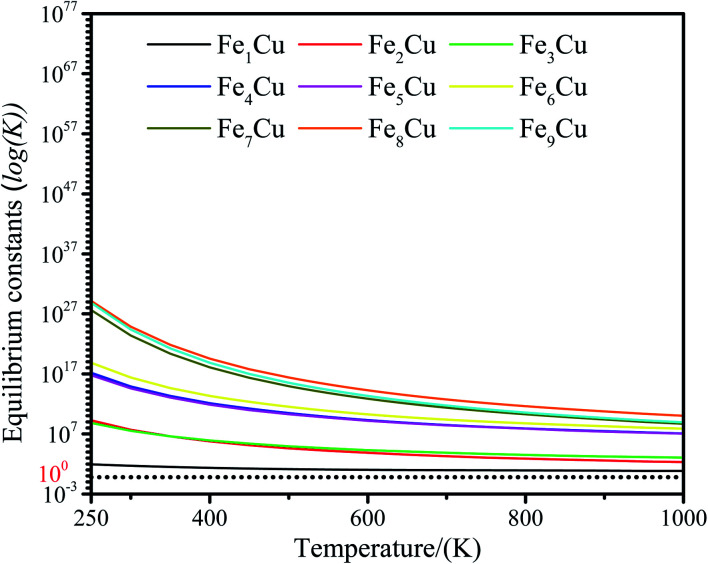
Equilibrium constants for CO_2_ decomposition on the Fe_*x*_/Cu(100) surface at temperature from 250 to 1000 K.

The zero point energy, entropy and enthalpy corrections for the activation energy barrier (*E*_a_) were considered to accurately describe the reaction at high temperatures (from 250 to 1000 K) in this section. The obtained results were presented in Tables S2–S19.† The forward and reverse rate constants of the elementary reaction steps for CO_2_ decomposition on the Fe_*x*_/Cu(100) surfaces at temperature ranging from 250 to 1000 K were displayed in Fig. 9. The Fig. 9 showed that the forward rate constants and inverse rate constants for the CO_2_ decomposition on the Fe_*x*_/Cu(100) surfaces increased as the temperature increased. At the same temperature, the rate constants for CO_2_ dissociation increased with an increase in the coverage of dopant Fe atom, indicating that the doped Fe significantly improved the rate constants. Moreover, it was further noting that the both forward rate constants and inverse rate constants for CO_2_ decomposition was not only increased as the increase of temperature, but the forward rate constants in all Fe_*x*_/Cu(100) surface were much larger than the inverse rate constants. In addition, the equilibrium constants (*K* = *k*_+_/*k*_−_) of CO_2_ decomposition on the Fe_*x*_/Cu(100) surfaces were also considered as a critical parameter. As shown in Fig. 10 and in Tables S11–S19,† the *K* decreased as the temperature increased, which meant that the increase in the inverse rate constants was greater than the positive rate constants as the temperature increased. The nine curves for *K* (equilibrium constants) were divided into four groups, *i.e.* G1 (Fe_1_/Cu system), G2 (Fe_2_/Cu and Fe_3_/Cu systems), G3 (Fe_4_/Cu, Fe_5_/Cu and Fe_6_/Cu systems), G4 (Fe_7_/Cu, Fe_8_/Cu and Fe_9_/Cu systems). In each group the equilibrium constants were close to each other, which was agreement with the variation in the BEP relationship. According to the *K*, the order of the four group was G4 > G3 > G2 > G1 at the same temperature. Thus, the doped Fe atom was favorable to be activation and decomposition of CO_2_ molecule and the *K* of CO_2_ decomposition increased as the coverage of the Fe atom increased. Moreover, although increasing temperature was favorable to the increases of the forward rate constants and invers rate constants, it was unfavorable to the increase of the equilibrium constants. From the perspective of dynamics, this results indicated that the lower the temperature for CO_2_ decomposition, the more favorable the decomposition of CO_2_ on the Fe_*x*_/Cu(100) surface.

#### 3.3.3. Effects of partial pressure of CO_2_

**Fig. 11:**
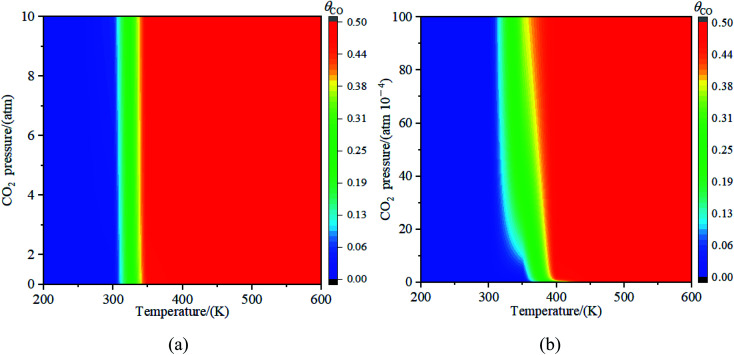
The concentration of CO on Fe_4_/Cu(100) surface with the partial pressure for CO_2_ (a) from 0 to 10 atm and (b) from 0 to 0.01 atm.

Fig. 11 illustrated the impact of partial pressure of CO_2_ towards the decomposition of CO_2_. The results in Fig. 11(a) showed that the decomposition of CO_2_ into CO at partial pressure from 0 to 10 atm was not obviously observed when the temperature was less than 300 K. While the concentration of CO reached 50% at the partial pressure of 0.5 atm when the temperature is higher than or equal to 300 K. To further explore the decomposition of CO_2_ at the low partial pressure of CO_2_, the partial pressure of CO_2_ from 0 atom to 0.01 atm was studied and the results were shown in Fig. 11(b). It was seen in Fig. 11(b) that when the temperature is below 300 K, the decomposition reaction of CO_2_ was not observed at the partial pressure from 0 atm to 0.01 atm; The relative concentration of CO increased with the partial pressure of CO_2_ increased when the temperature was at 350 K; if the temperature was greater than 350 K, the concentration of CO reached 49% at the partial pressure of 0.0003 atm (in general, the partial pressure of CO_2_ in the atmosphere is less than 0.03%). Therefore, when the temperature was greater than 350 K, CO_2_ could be decomposed into CO on Fe_4_/Cu(100) surface under the normal partial pressure of CO_2_ in the atmosphere.

### 3.4. Hydrogenation of CO_2_ on Fe_4_/Cu(100) surface

**Fig. 12:**
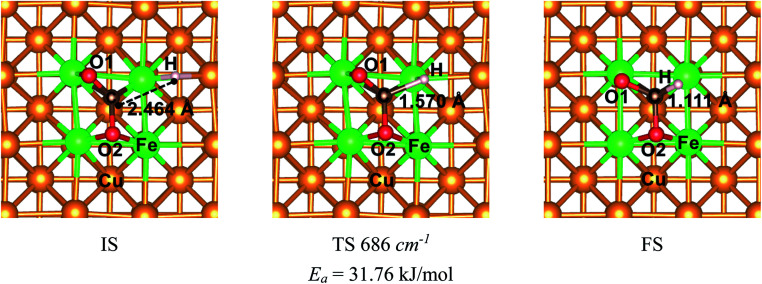
The structures of initial structure (IS), translation structure (TS) and final structure (FS) for CO_2_ hydrogenation to HCOO* on the Fe_4_/Cu(100) surface.

The mechanism of CO_2_ hydrogenation on the Fe_4_/Cu(100) surface was also examined in this section. There were three reaction pathways for CO_2_ hydrogenation: the C atom was attacked by H atom and the O1 and O2 atom was attacked, respectively. However, our previous works reveal that the hydrogenation of the C atom in CO_2_ moiety is more favorable than that of the O atom.^16,33,34^ Thus, the C atom hydrogenation was considered as an important reaction pathway while CO_2_ moiety was dissociated. The initial structure (IS), translation structure (TS) and finally structure (FS) was presented in Fig. 12. In Fig. 12, the distance between C and H atoms was changed from 2.464 Å in the IS to 1.570 Å in the TS, and then to 1.111 Å in the FS. The translation structure had been confirmed by the single imaginary frequency with 686 cm^−1^. It was worth noted that the calculated activation energy barrier of CO_2_ hydrogenation to HCOO* was 31.8 kJ mol^−1^, which was less 1.0 kJ mol^−1^ than that of decomposition for CO_2_ on the Fe_4_/Cu(100) surface. Such the small difference suggests that CO_2_ hydrogenation could also be performed during the CO_2_ decomposition. To understand the relationship between hydrogenation and dissociation of CO_2_, the FPMD was carried to the system with CO_2_ and H co-adsorption on Fe_4_/Cu(100) surface at temperature from 250 to 450 K. The radial distribution function of C–O suggests that the broad peak was observed from 2.5 to 3.5 Å in Fig. S15(a)† and it was not found for the peak of C–H in Fig. S15(b)† and O–H in Fig. S15(c)† from 1.0 to 1.5 Å at 400 K. This means that CO_2_ would rather decompose than hydrogenate at 400 K.

## 4. Conclusions

The adsorption, activation and reduction of CO_2_ molecule on the Fe_*x*_/Cu(100) (*x* = 1–9) had been investigated by the density functional theory based on the first principle. The results indicated that the introduction of dopant Fe atom could enhance the adsorption and activation of CO_2_ molecule on the Cu(100) surface. The most stable structure for CO_2_ on the Fe_*x*_/Cu(100) surface was sensitive to the coverage of the Fe dopant. The electronic structural analysis, including d-band center, Bader charge, vibrational frequencies, showed that CO_2_ molecule interacted with the β-state orbitals of the Fe_*x*_/Cu(100) surface. After CO_2_ adsorption, some electrons were transferred from substrate to CO_2_ moiety and the electrons transferred to CO_2_ moiety increased with the coverage of Fe atoms increased, leading to formation of CO_2_^−^ anion. Additionally, the mechanism of CO_2_ moiety decomposition on Fe_*x*_/Cu(100) surfaces had been studied in detail. The results indicated that the activation energy barrier (*E*_a_ = 32.8 kJ mol^−1^) of CO_2_ decomposition on Fe_4_/Cu(100) was the smallest among the Fe_*x*_/Cu(100) surfaces. The major reason was that, if coverage was more than 4/9 ML, the extra formed Fe–O2 bond played an handle role for CO_2_ decomposition. From the viewer of kinetic, the rate constants of CO_2_ on Fe_4_/Cu(100) surface were close to that of the Fe_9_/Cu(100) surface and the equilibrium constants analysis revealed that the servers of the equilibrium constants were divided into the four group, and the order of the four groups was G1 (Fe_1_/Cu) > G2 (Fe_2_/Cu and Fe_3_/Cu) > G3 (Fe_4_/Cu, Fe_5_/Cu and Fe_6_/Cu) > G4 (Fe_7_/Cu, Fe_8_/Cu and Fe_9_/Cu). Furthermore, our results confirmed that the lower the temperature, the more favorable it was to decompose the CO_2_ molecule into CO. When the simulated temperature was in range from 350 K and 450 K, the decomposition of C–O1 bond in CO_2_ moiety was only observed. The results of partial pressure for CO_2_ revealed that when the temperature was in range from 350 K to 450 K, the concentrate of CO on the Fe_4_/Cu(100) surface reached 49% under the partial pressure of 3 × 10^−4^ atm. Lastly, the mechanism of CO_2_ hydrogenation on the Fe_4_/Cu(100) surface was also investigated. The activation energy barrier of 31.8 kJ mol^−1^ was slightly less than that of CO_2_ decomposition. However, the results of the FPMD analysis revealed that CO_2_ was decomposed to form CO*, instead of hydrogenated. Our results provide insight into the mechanism for CO_2_ decomposition and hydrogenation on bimetallic surfaces from the perspective of thermodynamics and kinetics, which was important for the design and optimization of novel Cu-based bimetallic catalysts.

## Conflicts of interest

The authors declare that they have no known competing financial interests or personal relationships that could have appeared to influence the work reported in this paper.

## Supplementary Material

RA-010-D0RA06319C-s001

## References

[cit1] Kar S., Sen R., Goeppert A., Prakash G. S. (2018). J. Am. Chem. Soc..

[cit2] Gao G., Jiao Y., Waclawik E. R., Du A. (2016). J. Am. Chem. Soc..

[cit3] Torelli D. A., Francis S. A., Crompton J. C., Javier A., Thompson J. R., Brunschwig B. S., Soriaga M. P., Lewis N. S. (2016). ACS Catal..

[cit4] Cave E. R., Shi C., Kuhl K. P., Hatsukade T., Abram D. N., Hahn C., Chan K., Jaramillo T. F. (2018). ACS Catal..

[cit5] Ledezma-Yanez I., Gallent E. P., Koper M. T., Calle-Vallejo F. (2016). Catal. Today.

[cit6] Kumagai H., Nishikawa T., Koizumi H., Yatsu T., Sahara G., Yamazaki Y., Tamaki Y., Ishitani O. (2019). Chem. Sci..

[cit7] Pan F., Li B., Deng W., Du Z., Gang Y., Wang G., Li Y. (2019). Appl. Catal., B.

[cit8] Pi Y., Guo J., Shao Q., Huang X. (2019). Nano Energy.

[cit9] Li X., Bi W., Zhang L., Tao S., Chu W., Zhang Q., Luo Y., Wu C., Xie Y. (2016). Adv. Mater..

[cit10] Chen E.-X., Qiu M., Zhang Y.-F., Zhu Y.-S., Liu L.-Y., Sun Y.-Y., Bu X., Zhang J., Lin Q. (2018). Adv. Mater..

[cit11] Remiro-Buenamañana S., García H. (2018). ChemCatChem.

[cit12] Hartadi Y., Widmann D., Behm R. J. (2016). J. Catal..

[cit13] Qiu M., Tao H., Li R., Li Y., Huang X., Chen W., Su W., Zhang Y. (2016). J. Chem. Phys..

[cit14] Kuld S., Thorhauge M., Falsig H., Elkjær C. F., Helveg S., Chorkendorff I., Sehested J. (2016). Science.

[cit15] Gao P., Li S., Bu X., Dang S., Liu Z., Wang H., Zhong L., Qiu M., Yang C., Cai J., Wei W., Sun Y. (2017). Nat. Chem..

[cit16] Wagner T., Ermler U., Shima S. (2016). Science.

[cit17] Zheng Y., Zhang W. Q., Li Y. F., Chen J., Yu B., Wang J. C., Zhang L., Zhang J. J. (2017). Nano Energy.

[cit18] Zhao G. X., Huang X. B., Wang X. X., Wang X. K. (2017). J. Mater. Chem. A.

[cit19] Víctor A., González S., Illas F., Fierro J. L. (2008). Chem. Phys. Lett..

[cit20] Wang S.-G., Cao D.-B., Li Y.-W., Wang J., Jiao H. (2005). J. Phys. Chem. B.

[cit21] Farjamnia A., Jackson B. (2017). J. Chem. Phys..

[cit22] Rasmussen P., Taylor P., Chorkendorff I. (1992). Surf. Sci..

[cit23] Freund H.-J., Roberts M. W. (1996). Surf. Sci. Rep..

[cit24] Ding X., Pagan V., Peressi M., Ancilotto F. (2007). Mater. Sci. Eng., C.

[cit25] Glezakou V.-A., Dang L. X., McGrail B. P. (2009). J. Phys. Chem. C.

[cit26] Liu C., Cundari T. R., Wilson A. K. (2012). J. Phys. Chem. C.

[cit27] Ferrando R., Jellinek J., Johnston R. L. (2008). Chem. Rev..

[cit28] Steinhauer B., Kasireddy M. R., Radnik J., Martin A. (2009). Appl. Catal., A.

[cit29] Nerlov J., Chorkendorff I. (1999). J. Catal..

[cit30] Nerlov J., Sckerl S., Wambach J., Chorkendorff I. (2000). Appl. Catal., A.

[cit31] Nerlov J., Chorkendorff I. (1998). Catal. Lett..

[cit32] Yang Y., White M. G., Liu P. (2011). J. Phys. Chem. C.

[cit33] Qiu M., Tao H., Li Y., Li Y., Ding K., Huang X., Chen W., Zhang Y. (2018). Appl. Surf. Sci..

[cit34] Qiu M., Fang Z., Li Y., Zhu J., Huang X., Ding K., Chen W., Zhang Y. (2015). Appl. Surf. Sci..

[cit35] Nie X., Wang H., Liang Z., Yu Z., Zhang J., Janik M. J., Guo X., Song C. (2019). J. CO2 Util..

[cit36] Nie X., Wang H., Janik M. J., Guo X., Song C. (2016). J. Phys. Chem. C.

[cit37] Kurnosikov O., Kohlhepp J. T., de Jonge W. J. M. (2003). Europhys. Lett..

[cit38] Kolesnikov S. V., Klavsyuk A. L., Saletsky A. M. (2015). J. Exp. Theor. Phys..

[cit39] Kresse G., Hafner J. (1993). Phys. Rev. B: Condens. Matter Mater. Phys..

[cit40] Kresse G., Hafner J. (1993). Phys. Rev. B: Condens. Matter Mater. Phys..

[cit41] Kresse G., Hafner J. (1994). Phys. Rev. B: Condens. Matter Mater. Phys..

[cit42] Kresse G., Joubert D. (1999). Phys. Rev. B: Condens. Matter Mater. Phys..

[cit43] Blöchl P. E. (1994). Phys. Rev. B: Condens. Matter Mater. Phys..

[cit44] Hafner J. (2008). J. Comput. Chem..

[cit45] Perdew J. P., Burke K., Ernzerhof M. (1996). Phys. Rev. Lett..

[cit46] Grimme S. (2006). J. Comput. Chem..

[cit47] Ramalho J. P., Gomes J. R., Illas F. (2013). RSC Adv..

[cit48] Monkhorst H. J., Pack J. D. (1976). Phys. Rev. B: Solid State.

[cit49] Sheppard D., Xiao P., Chemelewski W., Johnson D. D., Henkelman G. (2012). J. Chem. Phys..

[cit50] Sheppard D., Terrell R., Henkelman G. (2008). J. Chem. Phys..

[cit51] Henkelman G., Jónsson H. (1999). J. Chem. Phys..

[cit52] Olsen R., Kroes G., Henkelman G., Arnaldsson A., Jónsson H. (2004). J. Chem. Phys..

[cit53] Henkelman G., Arnaldsson A., Jónsson H. (2006). Comput. Mater. Sci..

[cit54] Sanville E., Kenny S. D., Smith R., Henkelman G. (2007). J. Comput. Chem..

[cit55] Tang W., Sanville E., Henkelman G. (2009). J. Phys.: Condens. Matter.

[cit56] Eyring H. (1935). J. Chem. Phys..

[cit57] Nørskov J. K. , Studt F., Abild-Pedersen F. and Bligaard T., Fundamental Concepts in Heterogeneous Catalysis, Wiley, Hoboken, 2014, vol. 4, pp. 47–67

[cit58] Laidler K. J. , Chemical Kinetics, HarperCollins, New York, 3rd edn, 1987, vol. 4, pp. 81–129

[cit59] Bukoski A., Abbott H., Harrison I. (2005). J. Chem. Phys..

[cit60] Li K., Yin C., Zheng Y., He F., Wang Y., Jiao M., Tang H., Wu Z. (2016). J. Phys. Chem. C.

[cit61] Liu P., Rodriguez J. A. (2007). J. Chem. Phys..

[cit62] Novell-Leruth G., Ricart J. M., Pérez-Ramírez J. (2008). J. Phys. Chem. C.

[cit63] He C.-Z., Wang H., Huai L.-Y., Liu J.-Y. (2012). J. Phys. Chem. C.

