# Supplementary material for: The mechanism for CO2 reduction over Fe-modified Cu(100) surfaces with thermodynamics and kinetics: a DFT study
Source: RSC Adv. 2020 Sep 1;10(54):32569–80. doi: 10.1039/d0ra06319c (PMC9056627; doi:10.1039/d0ra06319c)
Supplement: RA-010-D0RA06319C-s001 [file RA-010-D0RA06319C-s001.pdf]

Electronic Supplementary Material (ESI) for RSC Advances.
This journal is © The Royal Society of Chemistry 2020

The mechanism for CO_2 reduction over Fe-modified Cu(100) surfaces with thermodynamics and kinetics: A DFT Study

Mei Qiu [a,b*], Yi Li [c], Yongfan Zhang [c]

Figure and Table Captions

[a] *Department of Chemistry, College of Science, Jiangxi Agricultural University, Nanchang, Jiangxi, 330045, China*
[b] *State Key Laboratory of Structural Chemistry, Fujian Institute of Research on the Structure of Matter, Chinese Academy of Sciences, 350002 Fuzhou, Fujian, 350002, China*
[c] *College of Chemistry, Fuzhou University, Fuzhou, Fujian, 350116, China*
To whom correspondence should be addressed. E-mail: qium@jxau.edu.cn (Mei Qiu)

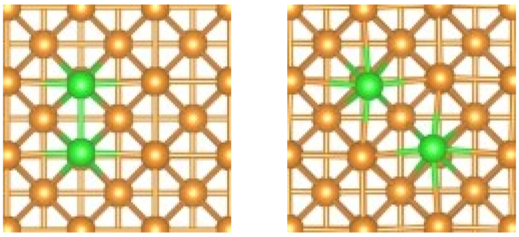

(a) M1(0 kJ/mol) (b) M2(28.83 kJ/mol)

$x = 2$

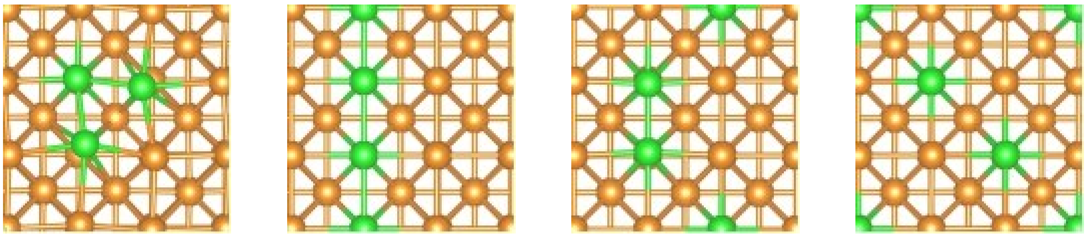

(a) M1(0 kcal/mol) (b) M2(11.46 kJ/mol) (c) M3(45.65 kJ/mol) (b) M4(73.22 kJ/mol)

$x = 3$

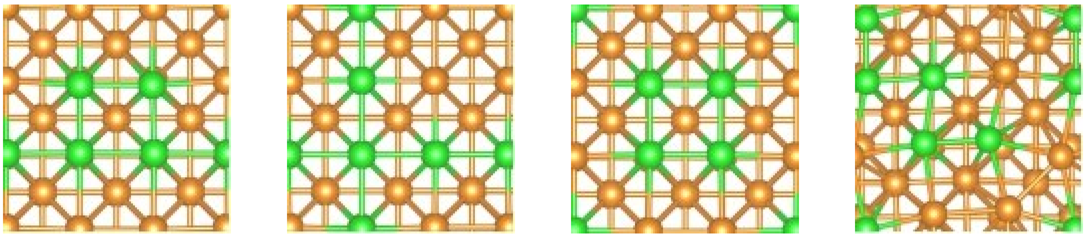

(a) M1(0 kJ/mol) (b) M2(21.67 kJ/mol) (c) M3(49.75 kJ/mol) (b) M4(102.42 kJ/mol)

$x = 5$

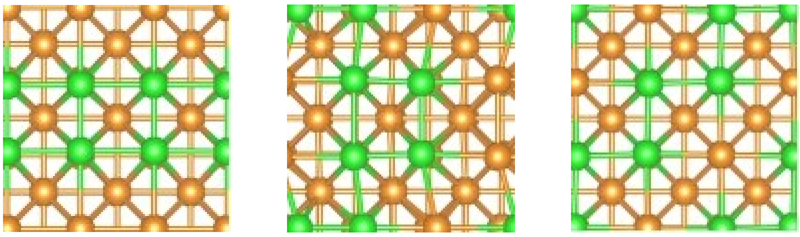

(a) M1(0 kJ/mol) (b) M2(21.21 kJ/mol) (c) M3(40.42 kJ/mol)

$x = 6$

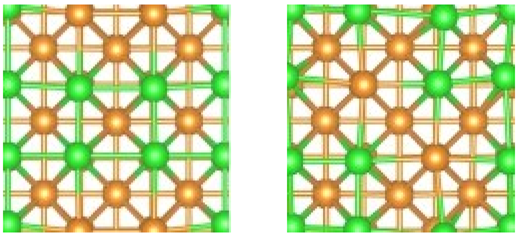

(a) M1(0 kJ/mol) (b) M2(34.43 kJ/mol)

$x = 7$

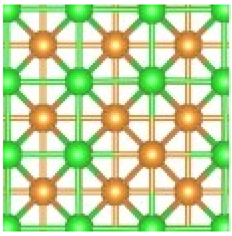

(a) M1

$x = 8$

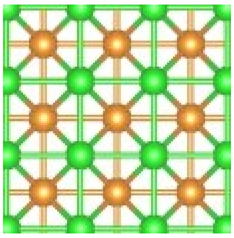

(a) M1

$x = 9$

Fig. S1 Top views and the relative energies (shown in parentheses) for Fe_x/Cu(100) (x = 2~3, 5 ~ 9) surface. Orange and green spheres stand for Cu and Fe atoms, respectively.

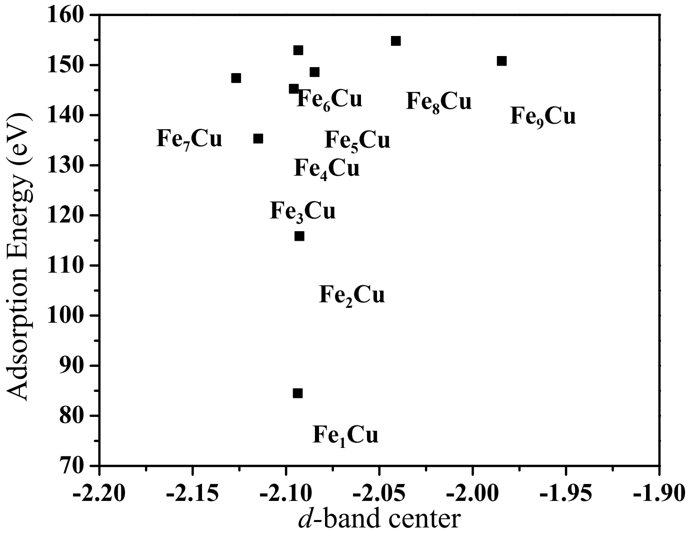

Fig. S2 Relationship between the *d*-band center for *alpha* orbitals of different Fe_x/Cu(100) surface and the adsorption energies of CO_2 molecule.

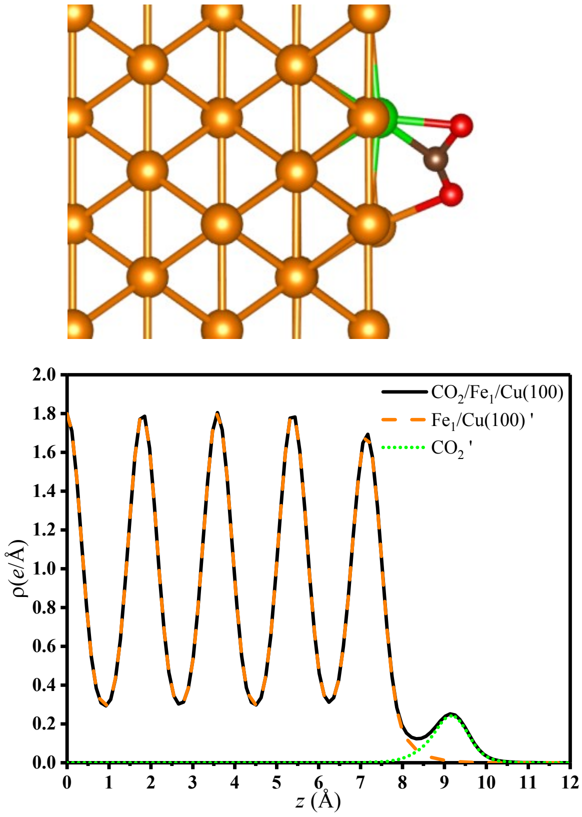

(a)

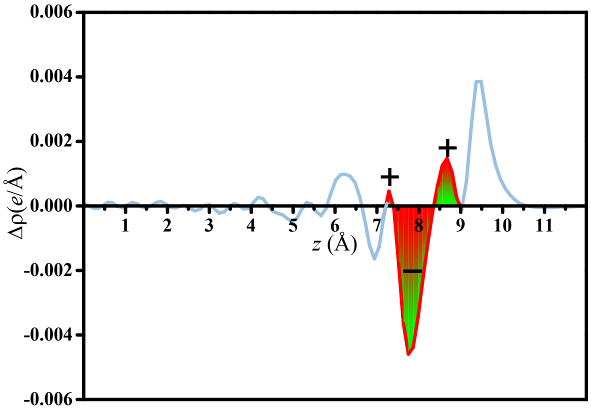

(b)

Fig. S3 (a) Charge density curve of isoluted Fe_1/Cu(10)′ surface, CO_2′ moiety and CO_2/Fe_1/Cu(100) surface. (b) Charge density difference curve of CO_2/Fe_1/Cu(100) surface: $\Delta\rho(z) = \rho_{CO_2/Fe_1/Cu(100)}(z) - \rho_{CO_2'}(z) - \rho_{Fe_1/Cu(100)'}(z)$. Fe_1/Cu(100) surface denotes from the CO_2/Fe_1/Cu(100) system and CO_2′ is the bent CO_2 moiety of the same CO_2/Fe_1/Cu(100) system.

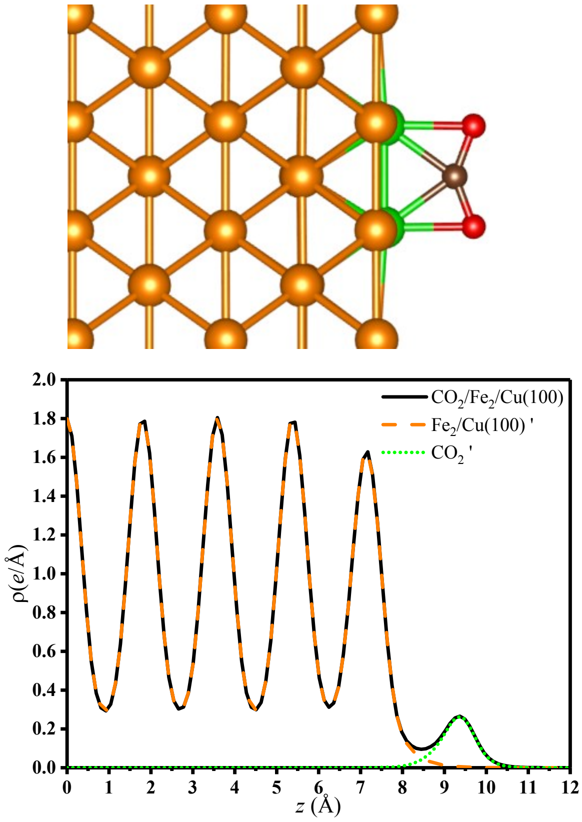

(a)

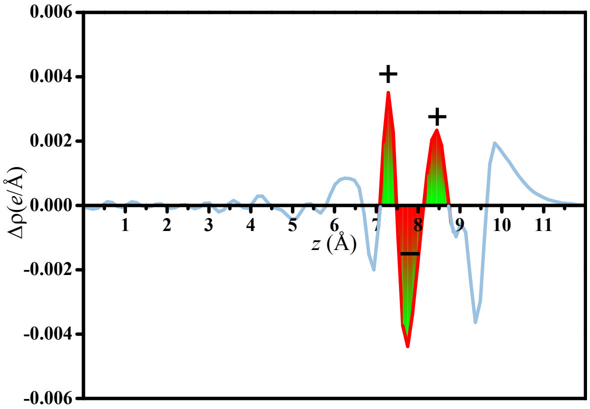

(b)

Fig. S4 (a) Charge density curve of isoluted Fe_2/Cu(10)′ surface, CO_2' moiety and CO_2/Fe_2/Cu(100) surface. (b) Charge density difference curve of CO_2/Fe_2/Cu(100) surface: $\Delta\rho(z) = \rho_{CO_2/Fe_2/Cu(100)}(z) - \rho_{CO_2'}(z) - \rho_{Fe_2/Cu(100)'}(z)$. Fe_2/Cu(100) surface denotes from the CO_2/Fe_2/Cu(100) system and CO_2' is the bent CO_2 moiety of the same CO_2/Fe_2/Cu(100) system.

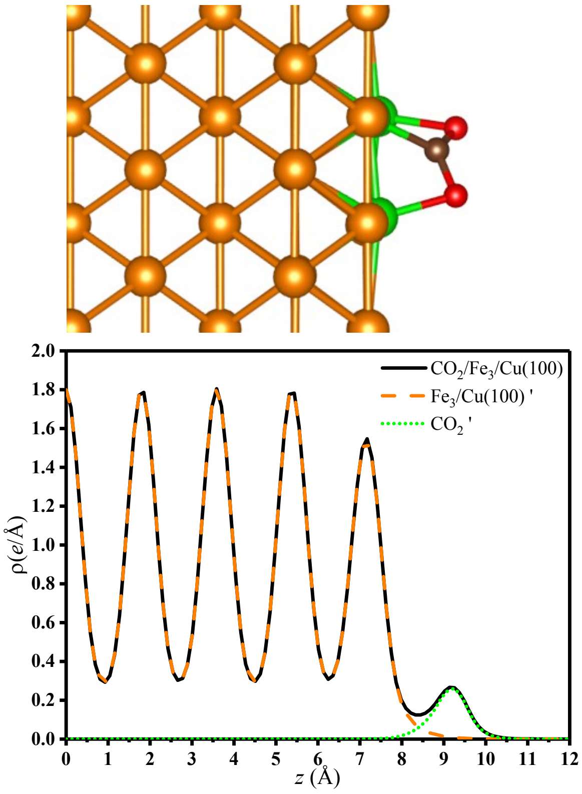

(a)

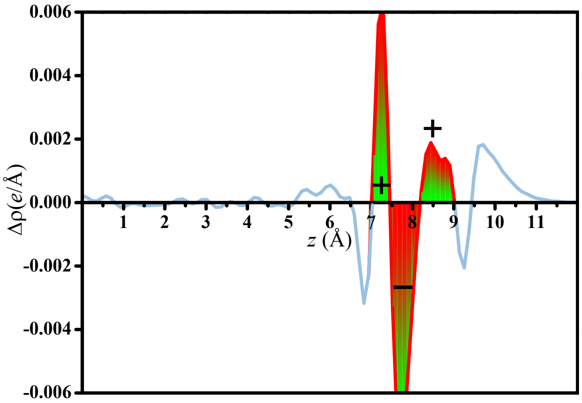

(b)

Fig. S5 (a) Charge density curve of isoluted $Fe_3/Cu(10)'$ surface, CO_2' moiety and $CO_2/Fe_3/Cu(100)$ surface. (b) Charge density difference curve of $CO_2/Fe_3/Cu(100)$ surface: $\Delta\rho(z) = \rho_{CO_2/Fe_3/Cu(100)}(z) - \rho_{CO_2'}(z) - \rho_{Fe_3/Cu(100)'}(z)$. $Fe_3/Cu(100)$ surface denotes from the $CO_2/Fe_3/Cu(100)$ system and CO_2' is the bent CO_2 moiety of the same $CO_2/Fe_3/Cu(100)$ system.

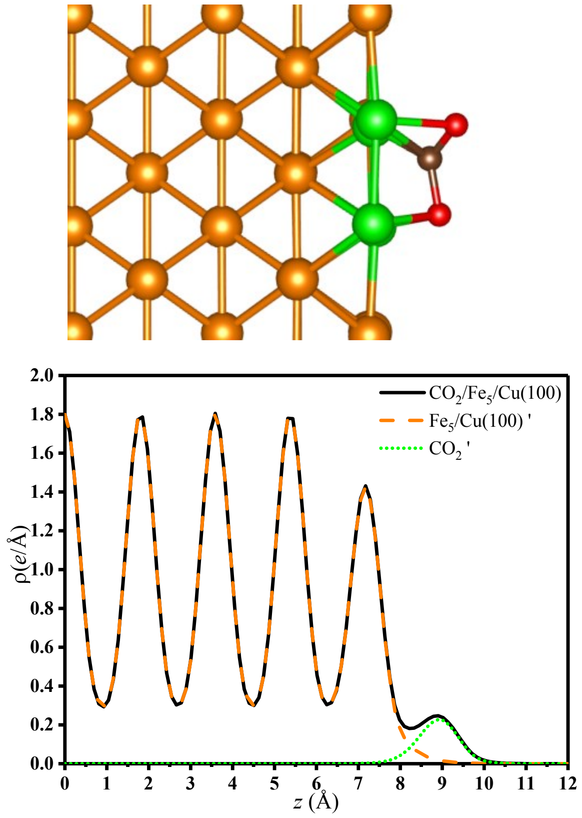

(a)

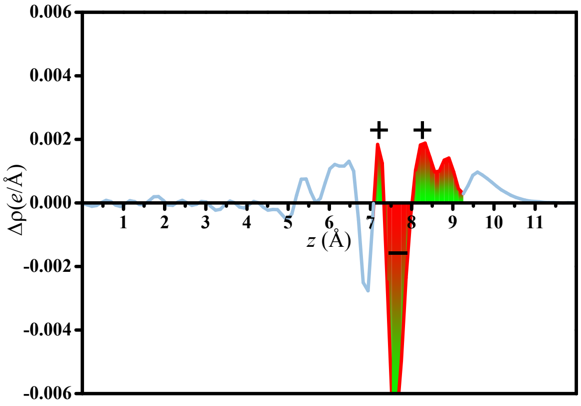

(b)

Fig. S6 (a) Charge density curve of isoluted $Fe_5/Cu(10)'$ surface, CO_2' moiety and $CO_2/Fe_5/Cu(100)$ surface. (b) Charge density difference curve of $CO_2/Fe_5/Cu(100)$ surface: $\Delta\rho(z) = \rho_{CO_2/Fe_5/Cu(100)}(z) - \rho_{CO_2'}(z) - \rho_{Fe_5/Cu(100)'}(z)$. $Fe_5/Cu(100)$ surface denotes from the $CO_2/Fe_5/Cu(100)$ system and CO_2' is the bent CO_2 moiety of the same $CO_2/Fe_5/Cu(100)$ system.

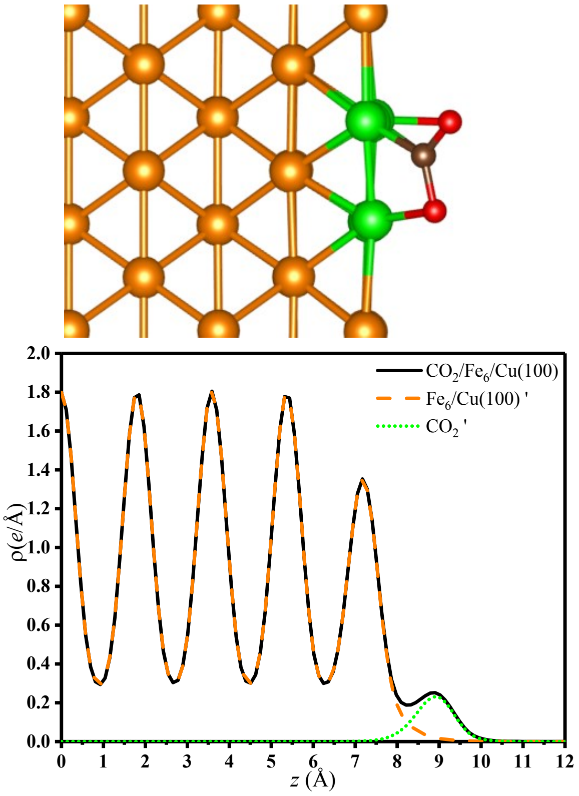

(a)

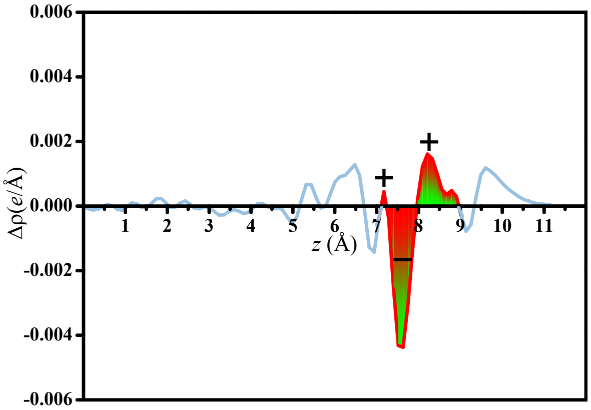

(b)

Fig. S7 (a) Charge density curve of isoluted $Fe_6/Cu(10)'$ surface, CO_2' moiety and $CO_2/Fe_6/Cu(100)$ surface. (b) Charge density difference curve of $CO_2/Fe_6/Cu(100)$ surface: $\Delta\rho(z) = \rho_{CO_2/Fe_6/Cu(100)}(z) - \rho_{CO_2'}(z) - \rho_{Fe_6/Cu(100)'}(z)$. $Fe_6/Cu(100)$ surface denotes from the $CO_2/Fe_6/Cu(100)$ system and CO_2' is the bent CO_2 moiety of the same $CO_2/Fe_6/Cu(100)$ system.

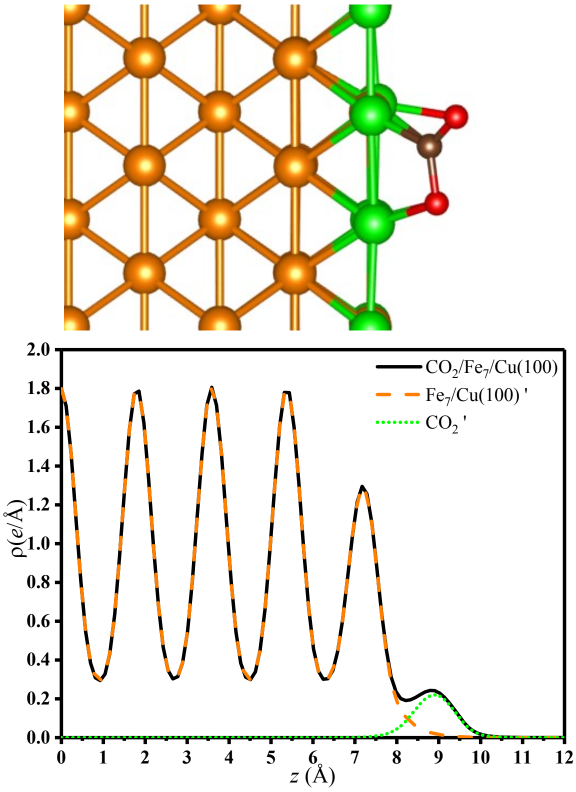

(a)

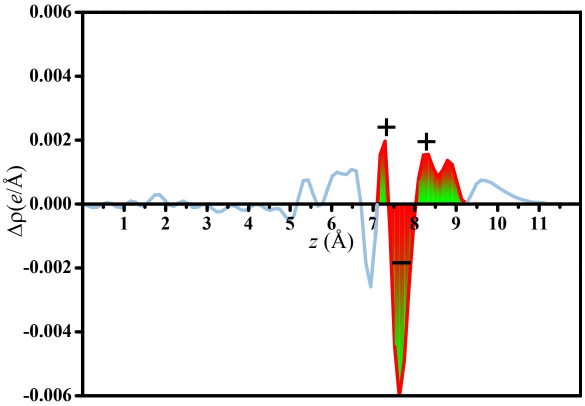

(b)

Fig. S8 (a) Charge density curve of isoluted $Fe_7/Cu(10)'$ surface, CO_2' moiety and $CO_2/Fe_7/Cu(100)$ surface. (b) Charge density difference curve of $CO_2/Fe_7/Cu(100)$ surface: $\Delta\rho(z) = \rho_{CO_2/Fe_7/Cu(100)}(z) - \rho_{CO_2'}(z) - \rho_{Fe_7/Cu(100)'}(z)$. $Fe_7/Cu(100)$ surface denotes from the $CO_2/Fe_7/Cu(100)$ system and CO_2' is the bent CO_2 moiety of the same $CO_2/Fe_7/Cu(100)$ system.

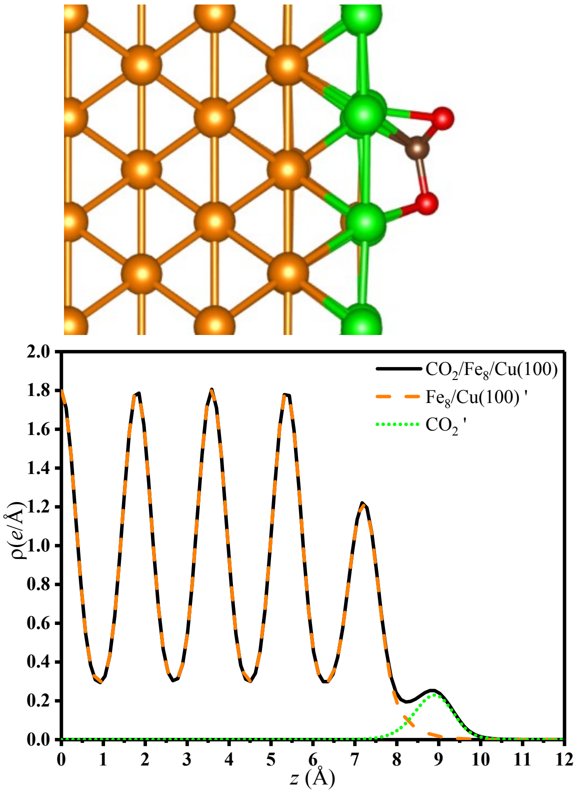

(a)

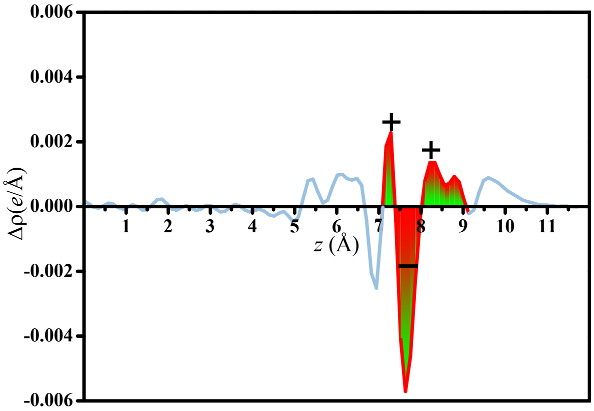

(b)

Fig. S9 (a) Charge density curve of isoluted $Fe_8/Cu(10)'$ surface, CO_2' moiety and $CO_2/Fe_8/Cu(100)$ surface. (b) Charge density difference curve of $CO_2/Fe_8/Cu(100)$ surface: $\Delta\rho(z) = \rho_{CO_2/Fe_8/Cu(100)}(z) - \rho_{CO_2'}(z) - \rho_{Fe_8/Cu(100)'}(z)$. $Fe_8/Cu(100)$ surface denotes from the $CO_2/Fe_8/Cu(100)$ system and CO_2' is the bent CO_2 moiety of the same $CO_2/Fe_8/Cu(100)$ system.

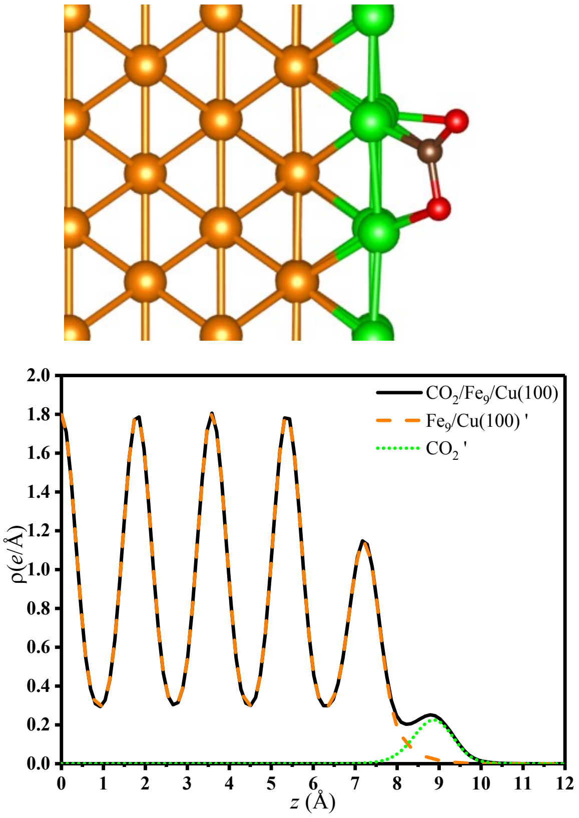

(a)

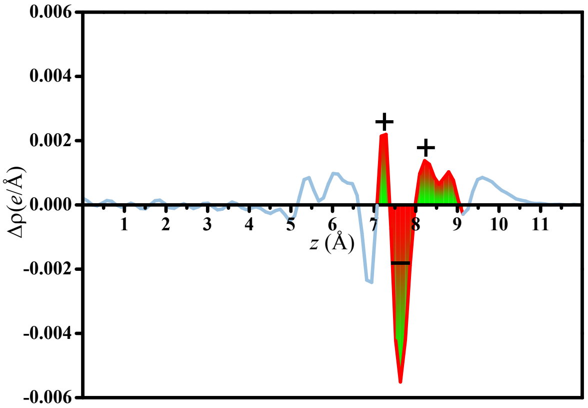

(b)

Fig. S10 (a) Charge density curve of isoluted $Fe_9/Cu(10)'$ surface, CO_2' moiety and $CO_2/Fe_9/Cu(100)$ surface. (b) Charge density difference curve of $CO_2/Fe_9/Cu(100)$ surface: $\Delta\rho(z) = \rho_{CO_2/Fe_9/Cu(100)}(z) - \rho_{CO_2'}(z) - \rho_{Fe_9/Cu(100)'}(z)$. $Fe_9/Cu(100)$ surface denotes from the $CO_2/Fe_9/Cu(100)$ system and CO_2' is the bent CO_2 moiety of the same $CO_2/Fe_9/Cu(100)$ system.

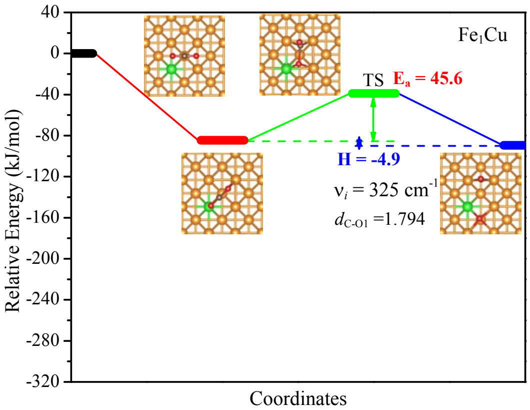
Fe1Cu
TS
Ea = 45.6
H = -4.9
νi = 325 cm-1
dC-O1 =1.794
Relative Energy (kJ/mol)
Coordinates

(a)

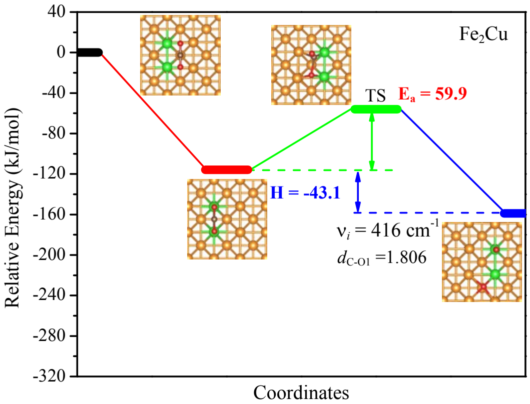
Fe2Cu
TS
Ea = 59.9
H = -43.1
νi = 416 cm-1
dC-O1 =1.806
Relative Energy (kJ/mol)
Coordinates

(b)

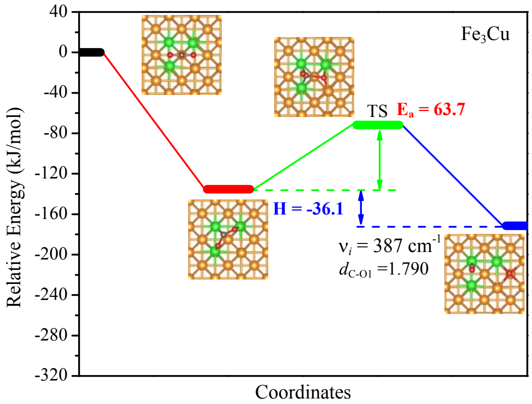
Fe3Cu
TS
Ea = 63.7
H = -36.1
νi = 387 cm-1
dC-O1 =1.790
Relative Energy (kJ/mol)
Coordinates

(c)

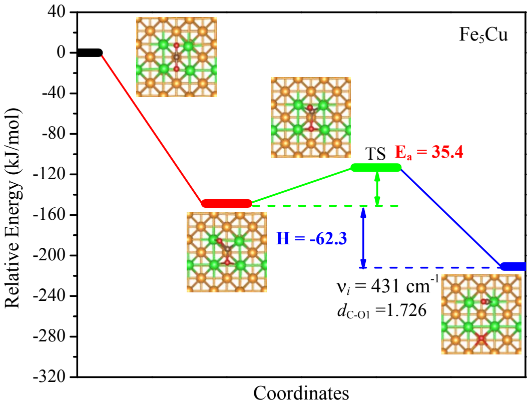
Fe5Cu
TS
Ea = 35.4
H = -62.3
νi = 431 cm-1
dC-O1 =1.726
Relative Energy (kJ/mol)
Coordinates

(d)

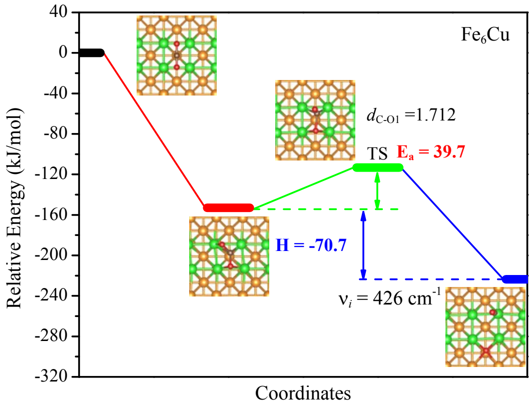
Fe6Cu
dC-O1 =1.712
TS
Ea = 39.7
H = -70.7
νi = 426 cm-1
Relative Energy (kJ/mol)
Coordinates

(e)

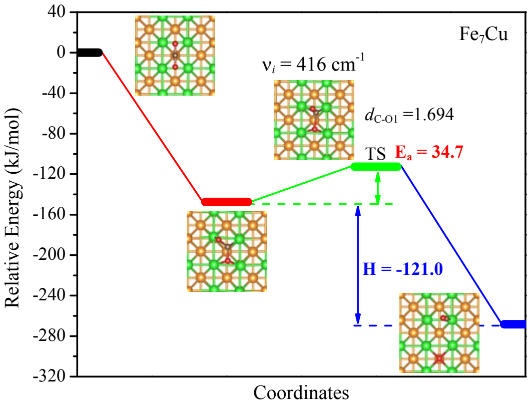
Fe7Cu
νi = 416 cm-1
dC-O1 =1.694
TS
Ea = 34.7
H = -121.0
Relative Energy (kJ/mol)
Coordinates

(f)

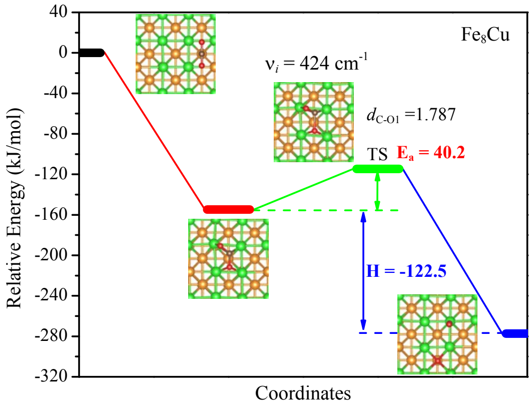
Fe8Cu
νi = 424 cm-1
dC-O1 =1.787
TS
Ea = 40.2
H = -122.5
Relative Energy (kJ/mol)
Coordinates

(g)

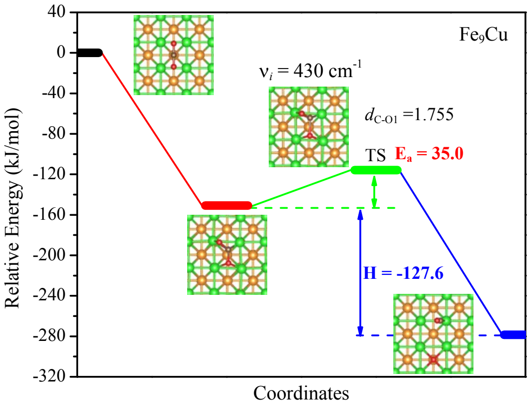
Fe9Cu
νi = 430 cm-1
dC-O1 =1.755
TS
Ea = 35.0
H = -127.6
Relative Energy (kJ/mol)
Coordinates

(h)

Fig. S11 Calculated reaction paths of CO_2 dissociation on the (a) Fe_1/Cu(100), (b) Fe_2/Cu(100), (c) Fe_3/Cu(100), (d) Fe_5/Cu(100), (e) Fe_6/Cu(100), (f) Fe_7/Cu(100), (g) Fe_8/Cu(100) and (h) Fe_9/Cu(100) surfaces. The zero of energy is set to the total energy of the isolated surface and CO_2 molecule in the gas phase.

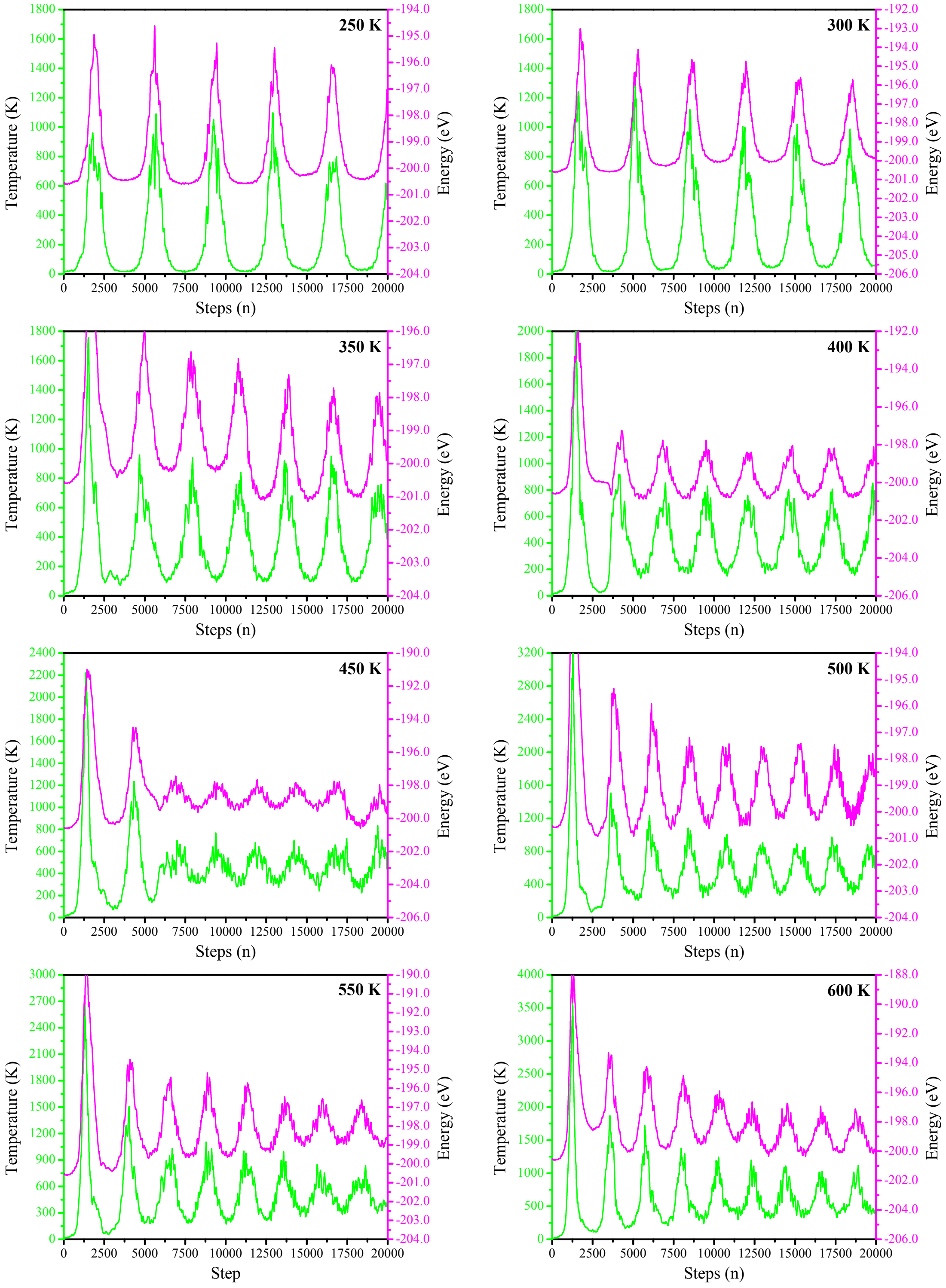
250 K
300 K
350 K
400 K
450 K
500 K
550 K
600 K
Temperature (K)
Energy (eV)
Steps (n)
Step

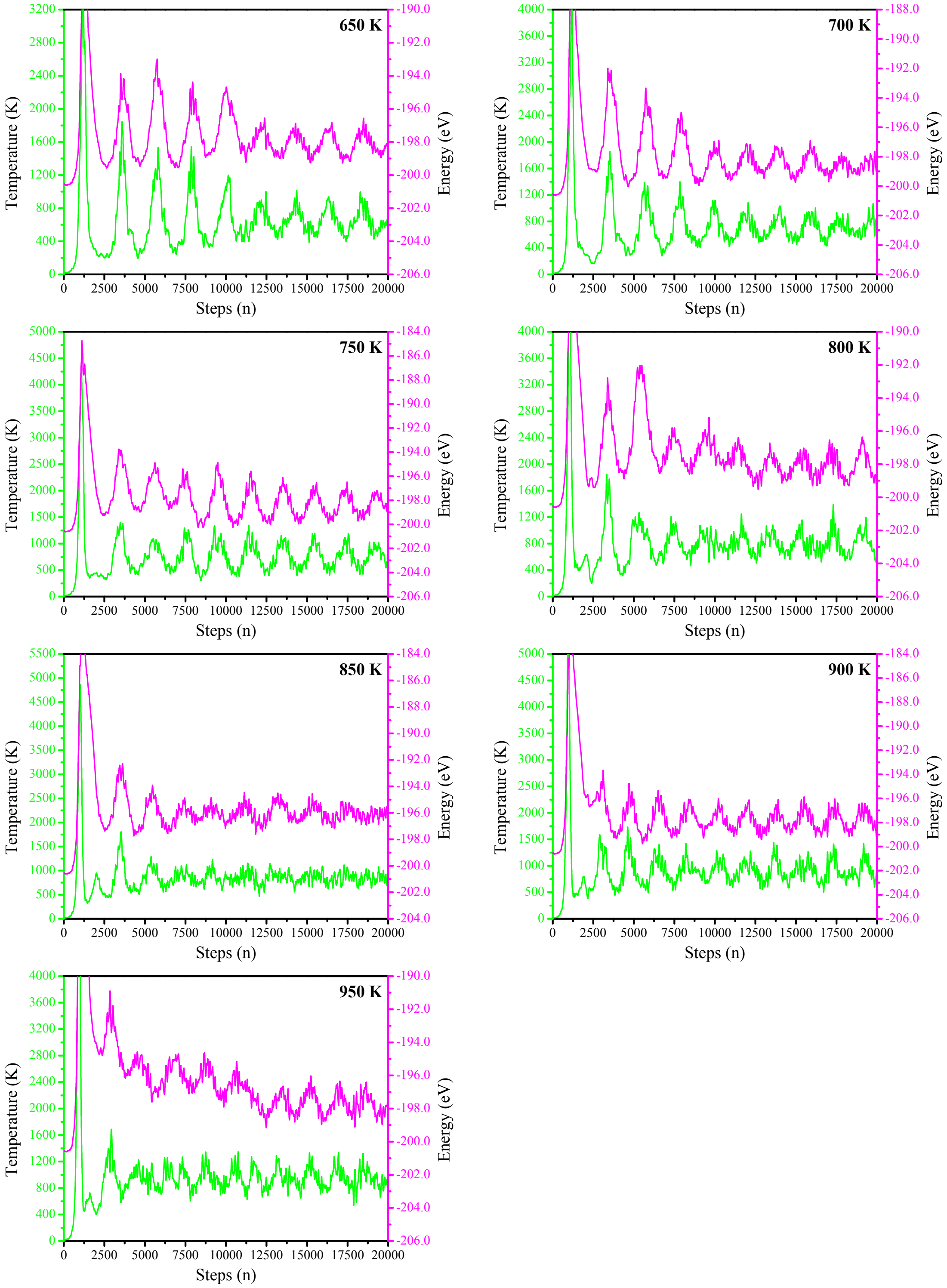

Fig. S12 the relationship between the temperature (K) and energy of the system per *fs* as the function of time steps at the simulated temperature from 250 to 950 K.

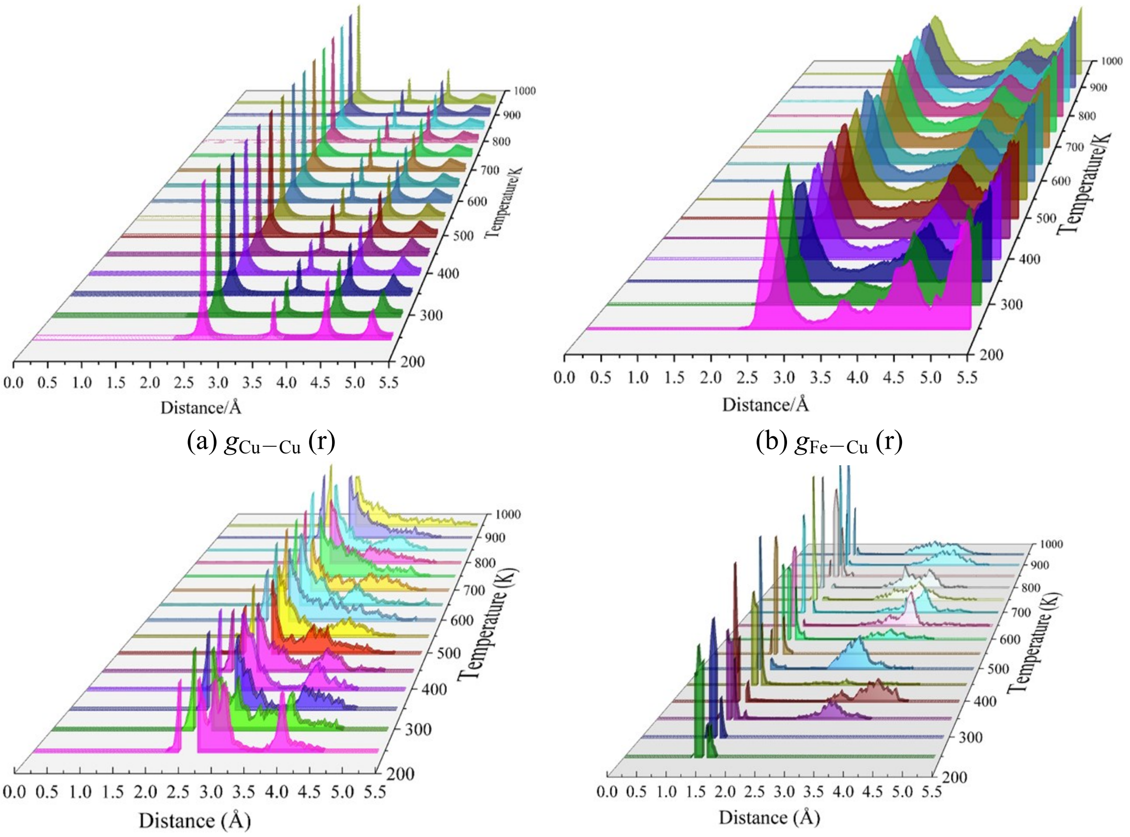

Fig. S13 The radial distribution function (g(r)) for (a) Cu－Cu, (b) Fe－Cu, (c) Fe－Fe and (d) C－O bonds in $CO_2/Fe_4/Cu(100)$ surface.

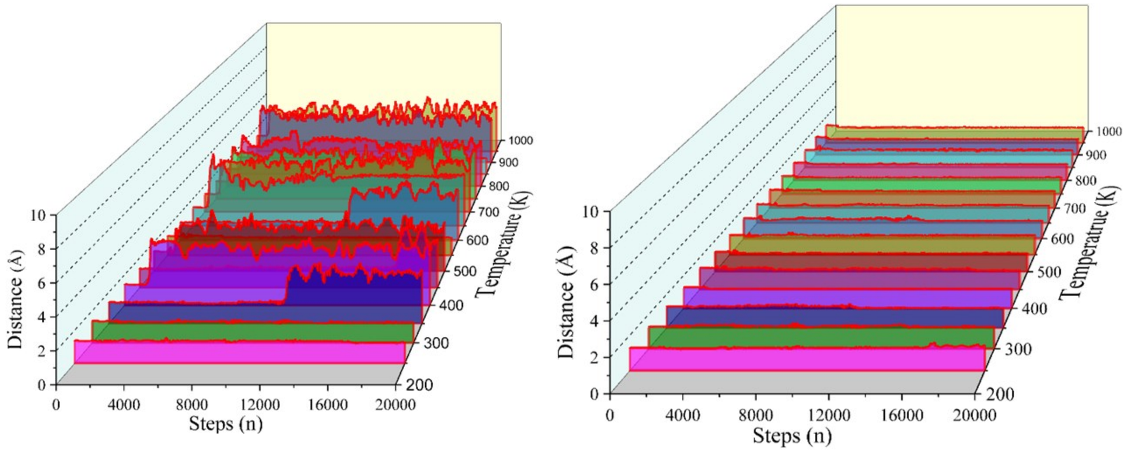

(a) (b)

Fig. S14 The distance of (a) C—O1 and (b) C—O2 bonds as the function of time steps at the simulated temperature from 250 to 950 K.

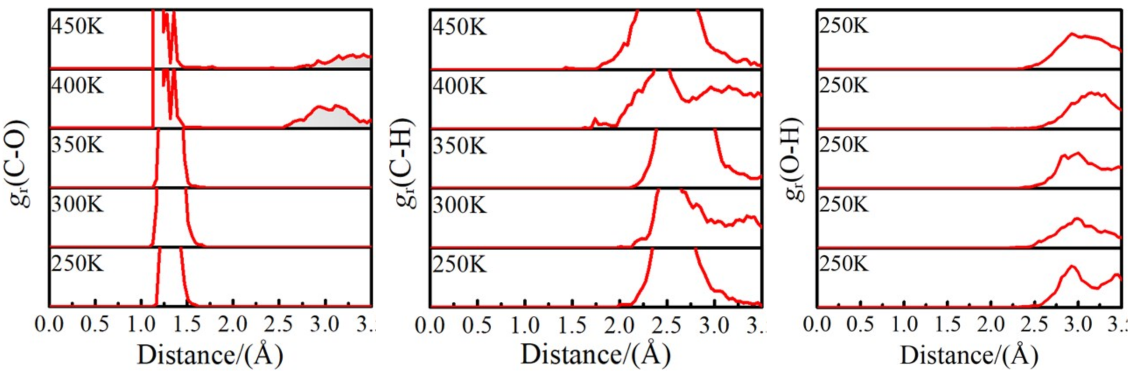

(a) (b) (c)

Fig. S15 The radial distribution function (g(r)) of the (a) C—O, (b) C—H, (c) O—H.

Table S1 Vibration frequencies (units: cm^{-1}) for the CO_2 moiety on the Fe_x/Cu(100) surface.

Model	Fe_1/Cu	Fe_2/Cu	Fe_3/Cu	Fe_4/Cu	Fe_5/Cu	Fe_6/Cu	Fe_7/Cu	Fe_8/Cu	Fe_9/Cu
1	1610	1707	1374	1305	1296	1283	1279	1258	1261
2	1122	1152	1117	1017	1019	1025	989	993	979
3	680	678	707	669	666	664	681	681	679
4	359	455	455	413	398	396	427	430	430
5	348	358	404	396	392	386	406	415	410
6	293	237	360	306	311	328	338	363	349
7	234	233	260	285	277	268	285	293	286
8	233	231	237	244	243	247	249	253	255
9	231	230	236	239	236	242	246	246	245

Table S2 The entropy and enthalpy corrections for energy barrier (E_a^+ and E_a^-) reaction energy (H^+ and H^-) for CO_2 decomposition on $Fe_1/Cu(100)$ surface at temperature from 250 to 1000 K. (Energy: kJ/mol Temperature: K)

Temperature	E_a^+		H^+		E_a^-		H^-	
	ΔTS	$\Delta\int C_p dT$	ΔTS	$\Delta\int C_p dT$	ΔTS	$\Delta\int C_p dT$	ΔTS	$\Delta\int C_p dT$
250	2.61	-0.74	0.24	0.69	2.37	-1.42	-0.24	-0.69
300	3.22	-0.82	-0.13	1.07	3.35	-1.89	0.13	-1.07
350	3.87	-0.92	-0.54	1.43	4.41	-2.35	0.54	-1.43
400	4.57	-1.06	-0.96	1.75	5.53	-2.81	0.96	-1.75
450	5.32	-1.23	-1.38	2.03	6.70	-3.26	1.38	-2.03
500	6.13	-1.44	-1.78	2.27	7.91	-3.70	1.78	-2.27
550	6.99	-1.67	-2.17	2.47	9.16	-4.14	2.17	-2.47
600	7.90	-1.94	-2.54	2.63	10.44	-4.57	2.54	-2.63
650	8.85	-2.22	-2.90	2.78	11.76	-5.00	2.90	-2.78
700	9.85	-2.53	-3.25	2.90	13.10	-5.43	3.25	-2.90
750	10.89	-2.85	-3.59	3.00	14.48	-5.85	3.59	-3.00
800	11.96	-3.18	-3.92	3.09	15.88	-6.27	3.92	-3.09
850	13.06	-3.52	-4.24	3.16	17.30	-6.69	4.24	-3.16
900	14.19	-3.88	-4.56	3.23	18.75	-7.11	4.56	-3.23
950	15.35	-4.24	-4.87	3.29	20.22	-7.53	4.87	-3.29
1000	16.53	-4.61	-5.18	3.34	21.71	-7.94	5.18	-3.34

Table S3 The entropy and enthalpy corrections for energy barrier (E_a^+ and E_a^-) reaction energy (H^+ and H^-) for CO_2 decomposition on Fe_2/Cu(100) surface at temperature from 250 to 1000 K. (Energy: kJ/mol Temperature: K)

Temperature	E_a^+		H^+		E_a^-		H^-	
	ΔTS	$\Delta\int C_p dT$	ΔTS	$\Delta\int C_p dT$	ΔTS	$\Delta\int C_p dT$	ΔTS	$\Delta\int C_p dT$
250	3.88	-1.12	0.76	-0.49	3.12	-0.63	-0.76	0.49
300	4.79	-1.24	0.75	-0.34	4.04	-0.90	-0.75	0.34
350	5.72	-1.37	0.69	-0.17	5.03	-1.20	-0.69	0.17
400	6.70	-1.51	0.60	0.01	6.10	-1.52	-0.60	-0.01
450	7.72	-1.69	0.50	0.18	7.22	-1.86	-0.50	-0.18
500	8.79	-1.88	0.40	0.33	8.39	-2.21	-0.40	-0.33
550	9.90	-2.11	0.30	0.46	9.60	-2.57	-0.30	-0.46
600	11.06	-2.36	0.21	0.57	10.85	-2.93	-0.21	-0.57
650	12.26	-2.62	0.13	0.67	12.13	-3.29	-0.13	-0.67
700	13.49	-2.91	0.05	0.75	13.44	-3.66	-0.05	-0.75
750	14.76	-3.20	-0.02	0.82	14.79	-4.03	0.02	-0.82
800	16.07	-3.52	-0.09	0.89	16.16	-4.40	0.09	-0.89
850	17.41	-3.84	-0.15	0.94	17.55	-4.78	0.15	-0.94
900	18.77	-4.17	-0.20	0.99	18.98	-5.16	0.20	-0.99
950	20.16	-4.51	-0.26	1.03	20.42	-5.54	0.26	-1.03
1000	21.58	-4.86	-0.31	1.06	21.89	-5.92	0.31	-1.06

Table S4 The entropy and enthalpy corrections for energy barrier (E_a^+ and E_a^-) reaction energy (H^+ and H^-) for CO_2 decomposition on Fe_3/Cu(100) surface at temperature from 250 to 1000 K. (Energy: kJ/mol Temperature: K)

Temperature	E_a^+		H^+		E_a^-		H^-	
	ΔTS	$\Delta\int C_p dT$	ΔTS	$\Delta\int C_p dT$	ΔTS	$\Delta\int C_p dT$	ΔTS	$\Delta\int C_p dT$
250	-1.34	0.50	-1.61	0.90	0.27	-0.40	1.61	-0.90
300	-1.66	0.54	-2.31	1.25	0.65	-0.70	2.31	-1.25
350	-1.92	0.53	-3.04	1.56	1.11	-1.03	3.04	-1.56
400	-2.12	0.46	-3.76	1.84	1.64	-1.38	3.76	-1.84
450	-2.25	0.33	-4.48	2.07	2.23	-1.74	4.48	-2.07
500	-2.31	0.15	-5.17	2.25	2.86	-2.10	5.17	-2.25
550	-2.31	-0.07	-5.84	2.40	3.54	-2.47	5.84	-2.40
600	-2.25	-0.33	-6.50	2.52	4.25	-2.85	6.5	-2.52
650	-2.15	-0.61	-7.14	2.61	4.99	-3.22	7.14	-2.61
700	-2.00	-0.91	-7.77	2.69	5.77	-3.60	7.77	-2.69
750	-1.81	-1.23	-8.38	2.75	6.57	-3.98	8.38	-2.75
800	-1.58	-1.57	-8.99	2.79	7.40	-4.36	8.99	-2.79
850	-1.32	-1.92	-9.58	2.83	8.26	-4.75	9.58	-2.83
900	-1.03	-2.28	-10.17	2.85	9.15	-5.13	10.17	-2.85
950	-0.71	-2.64	-10.76	2.87	10.05	-5.52	10.76	-2.87
1000	-0.37	-3.02	-11.34	2.89	10.98	-5.91	11.34	-2.89

Table S5 The entropy and enthalpy corrections for energy barrier (E_a^+ and E_a^-) reaction energy (H^+ and H^-) for CO_2 decomposition on Fe_4/Cu(100) surface at temperature from 250 to 1000 K. (Energy: kJ/mol Temperature: K)

Temperature	E_a^+		H^+		E_a^-		H^-	
	ΔTS	$\Delta\int C_p dT$	ΔTS	$\Delta\int C_p dT$	ΔTS	$\Delta\int C_p dT$	ΔTS	$\Delta\int C_p dT$
250	-2.78	0.80	-8.11	2.10	5.34	-1.30	8.11	-2.10
300	-3.32	0.78	-10.08	2.41	6.76	-1.63	10.08	-2.41
350	-3.78	0.70	-12.05	2.68	8.27	-1.98	12.05	-2.68
400	-4.17	0.56	-14.01	2.90	9.84	-2.34	14.01	-2.90
450	-4.48	0.36	-15.94	3.07	11.46	-2.71	15.94	-3.07
500	-4.72	0.12	-17.85	3.20	13.12	-3.08	17.85	-3.20
550	-4.90	-0.16	-19.73	3.29	14.82	-3.45	19.73	-3.29
600	-5.03	-0.47	-21.59	3.35	16.56	-3.83	21.59	-3.35
650	-5.10	-0.81	-23.43	3.40	18.33	-4.20	23.43	-3.40
700	-5.13	-1.16	-25.26	3.42	20.13	-4.58	25.26	-3.42
750	-5.12	-1.52	-27.08	3.43	21.96	-4.96	27.08	-3.43
800	-5.07	-1.90	-28.89	3.44	23.82	-5.33	28.89	-3.44
850	-4.99	-2.28	-30.69	3.43	25.70	-5.71	30.69	-3.43
900	-4.88	-2.67	-32.48	3.42	27.60	-6.10	32.48	-3.42
950	-4.75	-3.07	-34.27	3.41	29.53	-6.48	34.27	-3.41
1000	-4.59	-3.47	-36.06	3.39	31.48	-6.86	36.06	-3.39

Table S6 The entropy and enthalpy corrections for energy barrier (E_a^+ and E_a^-) reaction energy (H^+ and H^-) for CO_2 decomposition on Fe_5/Cu(100) surface at temperature from 250 to 1000 K. (Energy: kJ/mol Temperature: K)

Temperature	E_a^+		H^+		E_a^-		H^-	
	ΔTS	$\Delta\int C_p dT$	ΔTS	$\Delta\int C_p dT$	ΔTS	$\Delta\int C_p dT$	ΔTS	$\Delta\int C_p dT$
250	-2.85	0.75	-8.18	2.28	5.33	-1.53	8.18	-2.28
300	-3.39	0.73	-10.21	2.64	6.82	-1.92	10.21	-2.64
350	-3.85	0.63	-12.24	2.95	8.39	-2.31	12.24	-2.95
400	-4.24	0.48	-14.26	3.20	10.02	-2.71	14.26	-3.20
450	-4.56	0.28	-16.25	3.39	11.69	-3.11	16.25	-3.39
500	-4.80	0.03	-18.21	3.54	13.41	-3.51	18.21	-3.54
550	-4.98	-0.25	-20.15	3.65	15.16	-3.91	20.15	-3.65
600	-5.11	-0.57	-22.07	3.73	16.95	-4.30	22.07	-3.73
650	-5.19	-0.90	-23.97	3.79	18.78	-4.69	23.97	-3.79
700	-5.22	-1.25	-25.85	3.83	20.63	-5.08	25.85	-3.83
750	-5.22	-1.62	-27.72	3.86	22.50	-5.48	27.72	-3.86
800	-5.18	-1.99	-29.59	3.87	24.41	-5.87	29.59	-3.87
850	-5.11	-2.38	-31.44	3.88	26.34	-6.26	31.44	-3.88
900	-5.00	-2.77	-33.29	3.88	28.29	-6.65	33.29	-3.88
950	-4.88	-3.17	-35.14	3.88	30.26	-7.04	35.14	-3.88
1000	-4.72	-3.57	-36.98	3.87	32.26	-7.43	36.98	-3.87

Table S7 The entropy and enthalpy corrections for energy barrier (E_a^+ and E_a^-) reaction energy (H^+ and H^-) for CO_2 decomposition on Fe_6/Cu(100) surface at temperature from 250 to 1000 K. (Energy: kJ/mol Temperature: K)

Temperature	E_a^+		H^+		E_a^-		H^-	
	ΔTS	$\Delta\int C_p dT$	ΔTS	$\Delta\int C_p dT$	ΔTS	$\Delta\int C_p dT$	ΔTS	$\Delta\int C_p dT$
250	-3.75	0.80	-9.32	2.09	5.56	-1.29	9.32	-2.09
300	-4.47	0.77	-11.51	2.40	7.04	-1.62	11.51	-2.40
350	-5.12	0.68	-13.71	2.66	8.59	-1.97	13.71	-2.66
400	-5.69	0.53	-15.9	2.87	10.21	-2.34	15.9	-2.87
450	-6.19	0.33	-18.06	3.03	11.87	-2.71	18.06	-3.03
500	-6.61	0.08	-20.19	3.15	13.58	-3.08	20.19	-3.15
550	-6.97	-0.21	-22.30	3.24	15.33	-3.45	22.3	-3.24
600	-7.28	-0.52	-24.39	3.30	17.11	-3.82	24.39	-3.30
650	-7.54	-0.86	-26.46	3.33	18.93	-4.20	26.46	-3.33
700	-7.75	-1.21	-28.52	3.36	20.77	-4.57	28.52	-3.36
750	-7.92	-1.58	-30.57	3.36	22.64	-4.94	30.57	-3.36
800	-8.06	-1.96	-32.60	3.36	24.54	-5.32	32.6	-3.36
850	-8.17	-2.35	-34.63	3.35	26.47	-5.70	34.63	-3.35
900	-8.24	-2.74	-36.66	3.34	28.41	-6.08	36.66	-3.34
950	-8.29	-3.13	-38.68	3.33	30.38	-6.46	38.68	-3.33
1000	-8.32	-3.54	-40.69	3.31	32.37	-6.84	40.69	-3.31

Table S8 The entropy and enthalpy corrections for energy barrier (E_a^+ and E_a^-) reaction energy (H^+ and H^-) for CO_2 decomposition on Fe_7/Cu(100) surface at temperature from 250 to 1000 K. (Energy: kJ/mol Temperature: K)

Temperature	E_a^+		H^+		E_a^-		H^-	
	ΔTS	$\Delta\int C_p dT$	ΔTS	$\Delta\int C_p dT$	ΔTS	$\Delta\int C_p dT$	ΔTS	$\Delta\int C_p dT$
250	-1.46	0.52	-4.84	1.52	3.38	-1.00	4.84	-1.52
300	-1.74	0.51	-6.19	1.86	4.45	-1.36	6.19	-1.86
350	-1.95	0.43	-7.54	2.16	5.59	-1.73	7.54	-2.16
400	-2.07	0.28	-8.87	2.40	6.80	-2.11	8.87	-2.40
450	-2.12	0.08	-10.18	2.59	8.06	-2.50	10.18	-2.59
500	-2.10	-0.16	-11.46	2.73	9.36	-2.89	11.46	-2.73
550	-2.01	-0.45	-12.71	2.83	10.70	-3.27	12.71	-2.83
600	-1.86	-0.76	-13.94	2.90	12.08	-3.66	13.94	-2.90
650	-1.67	-1.10	-15.15	2.95	13.49	-4.05	15.15	-2.95
700	-1.43	-1.45	-16.35	2.98	14.92	-4.43	16.35	-2.98
750	-1.15	-1.82	-17.54	3.00	16.39	-4.82	17.54	-3.00
800	-0.83	-2.2	-18.72	3.01	17.88	-5.21	18.72	-3.01
850	-0.48	-2.59	-19.89	3.01	19.40	-5.60	19.89	-3.01
900	-0.11	-2.98	-21.05	3.00	20.94	-5.98	21.05	-3.00
950	0.30	-3.38	-22.21	2.99	22.51	-6.37	22.21	-2.99
1000	0.73	-3.78	-23.37	2.98	24.09	-6.77	23.37	-2.98

Table S9 The entropy and enthalpy corrections for energy barrier (E_a^+ and E_a^-) reaction energy (H^+ and H^-) for CO_2 decomposition on $Fe_8/Cu(100)$ surface at temperature from 250 to 1000 K. (Energy: kJ/mol Temperature: K)

Temperature	E_a^+		H^+		E_a^-		H^-	
	ΔTS	$\Delta\int C_p dT$	ΔTS	$\Delta\int C_p dT$	ΔTS	$\Delta\int C_p dT$	ΔTS	$\Delta\int C_p dT$
250	-2.37	0.61	-7.92	1.90	5.55	-1.29	7.92	-1.90
300	-2.80	0.58	-9.89	2.25	7.08	-1.68	9.89	-2.25
350	-3.17	0.48	-11.86	2.55	8.69	-2.07	11.86	-2.55
400	-3.46	0.33	-13.80	2.79	10.34	-2.46	13.80	-2.79
450	-3.68	0.13	-15.72	2.97	12.05	-2.85	15.72	-2.97
500	-3.83	-0.12	-17.61	3.11	13.78	-3.23	17.61	-3.11
550	-3.92	-0.40	-19.48	3.20	15.56	-3.60	19.48	-3.20
600	-3.96	-0.70	-21.32	3.27	17.36	-3.97	21.32	-3.27
650	-3.95	-1.03	-23.14	3.31	19.19	-4.34	23.14	-3.31
700	-3.90	-1.37	-24.94	3.33	21.04	-4.70	24.94	-3.33
750	-3.81	-1.73	-26.73	3.34	22.92	-5.07	26.73	-3.34
800	-3.69	-2.09	-28.51	3.34	24.83	-5.43	28.51	-3.34
850	-3.53	-2.46	-30.29	3.34	26.76	-5.80	30.29	-3.34
900	-3.35	-2.84	-32.06	3.32	28.71	-6.17	32.06	-3.32
950	-3.14	-3.23	-33.82	3.31	30.68	-6.54	33.82	-3.31
1000	-2.90	-3.62	-35.58	3.29	32.68	-6.91	35.58	-3.29

Table S10 The entropy and enthalpy corrections for energy barrier (E_a^+ and E_a^-) reaction energy (H^+ and H^-) for CO_2 decomposition on Fe_9/Cu(100) surface at temperature from 250 to 1000 K. (Energy: kJ/mol Temperature: K)

Temperature	E_a^+		H^+		E_a^-		H^-	
	ΔTS	$\Delta\int C_p dT$	ΔTS	$\Delta\int C_p dT$	ΔTS	$\Delta\int C_p dT$	ΔTS	$\Delta\int C_p dT$
250	-4.36	0.78	-4.52	1.62	0.17	-0.84	4.52	-1.62
300	-5.19	0.74	-5.82	1.98	0.64	-1.24	5.82	-1.98
350	-5.94	0.64	-7.12	2.28	1.18	-1.64	7.12	-2.28
400	-6.63	0.49	-8.40	2.53	1.78	-2.04	8.40	-2.53
450	-7.24	0.28	-9.65	2.72	2.42	-2.44	9.65	-2.72
500	-7.78	0.03	-10.88	2.86	3.10	-2.83	10.88	-2.86
550	-8.26	-0.25	-12.07	2.96	3.81	-3.21	12.07	-2.96
600	-8.69	-0.56	-13.24	3.03	4.56	-3.60	13.24	-3.03
650	-9.07	-0.89	-14.40	3.08	5.33	-3.97	14.40	-3.08
700	-9.41	-1.24	-15.53	3.11	6.13	-4.35	15.53	-3.11
750	-9.71	-1.60	-16.66	3.12	6.96	-4.72	16.66	-3.12
800	-9.97	-1.97	-17.78	3.13	7.81	-5.10	17.78	-3.13
850	-10.21	-2.35	-18.89	3.13	8.68	-5.48	18.89	-3.13
900	-10.41	-2.73	-19.99	3.12	9.58	-5.85	19.99	-3.12
950	-10.59	-3.12	-21.09	3.11	10.50	-6.23	21.09	-3.11
1000	-10.74	-3.51	-22.19	3.10	11.44	-6.61	22.19	-3.10

Table S11 The forward and inverse corrected activatin energy barrier (ΔE_a^+ and ΔE_a^-) and equilibrium constants (k_+ , k_- and K) for CO_2 on Fe_1/Cu(100) surface at temperature from 250 to 1000 K. (Energy: kJ/mol Temperature: K k: s^{-1})

Temperature (K)	ΔE_a^+	ΔE_a^-	k_+	k_-	$K(k_+/k_-)$
250	42.98	50.93	8.50×10^{3}	9.54×10^{1}	8.91×10^{1}
300	43.51	51.45	2.16×10^{5}	4.60×10^{3}	4.69×10^{1}
350	44.06	52.04	2.16×10^{6}	7.14×10^{4}	3.03×10^{1}
400	44.62	52.70	1.21×10^{7}	5.47×10^{5}	2.22×10^{1}
450	45.20	53.42	4.61×10^{7}	2.63×10^{6}	1.75×10^{1}
500	45.80	54.19	1.34×10^{8}	9.11×10^{6}	1.47×10^{1}
550	46.42	55.00	3.17×10^{8}	2.49×10^{7}	1.27×10^{1}
600	47.07	55.85	6.49×10^{8}	5.73×10^{7}	1.13×10^{1}
650	47.74	56.74	1.19×10^{9}	1.15×10^{8}	1.03×10^{1}
700	48.43	57.66	1.98×10^{9}	2.08×10^{8}	9.51×10^{0}
750	49.15	58.61	3.07×10^{9}	3.46×10^{8}	8.89×10^{0}
800	49.88	59.59	4.50×10^{9}	5.37×10^{8}	8.38×10^{0}
850	50.64	60.60	6.28×10^{9}	7.89×10^{8}	7.97×10^{0}
900	51.42	61.63	8.43×10^{9}	1.11×10^{9}	7.62×10^{0}
950	52.22	62.68	1.09×10^{10}	1.49×10^{9}	7.32×10^{0}
1000	53.04	63.75	1.38×10^{10}	1.95×10^{9}	7.07×10^{0}

Table S12 The forward and inverse corrected activatin energy barrier (ΔE_a^+ and ΔE_a^-) and equilibrium constants (k_+ , k_- and K) for CO_2 on Fe_2/Cu(100) surface at temperature from 250 to 1000 K. (Energy: kJ/mol Temperature: K k: s^{-1})

Temperature	ΔE_a^+	ΔE_a^-	k_+	k_-	$K(k_+/k_-)$
250	57.83	102.15	3.79×10^{0}	1.95×10^{-9}	1.95×10^{9}
300	58.62	102.79	2.85×10^{2}	5.41×10^{-6}	5.27×10^{7}
350	59.42	103.49	6.20×10^{3}	1.54×10^{-3}	4.04×10^{6}
400	60.25	104.23	6.20×10^{4}	1.05×10^{-1}	5.92×10^{5}
450	61.10	105.01	3.70×10^{5}	2.76×10^{0}	1.34×10^{5}
500	61.97	105.83	1.54×10^{6}	3.76×10^{1}	4.09×10^{4}
550	62.86	106.69	4.91×10^{6}	3.15×10^{2}	1.56×10^{4}
600	63.77	107.58	1.29×10^{7}	1.84×10^{3}	6.98×10^{3}
650	64.70	108.50	2.89×10^{7}	8.17×10^{3}	3.54×10^{3}
700	65.65	109.44	5.78×10^{7}	2.91×10^{4}	1.98×10^{3}
750	66.63	110.42	1.05×10^{8}	8.73×10^{4}	1.20×10^{3}
800	67.62	111.41	1.76×10^{8}	2.27×10^{5}	7.74×10^{2}
850	68.63	112.43	2.77×10^{8}	5.27×10^{5}	5.26×10^{2}
900	69.67	113.48	4.14×10^{8}	1.11×10^{6}	3.73×10^{2}
950	70.72	114.54	5.92×10^{8}	2.15×10^{6}	2.75×10^{2}
1000	71.79	115.63	8.14×10^{8}	3.90×10^{6}	2.09×10^{2}

Table S13 The forward and inverse corrected activatin energy barrier (ΔE_a^{+} and ΔE_a^{-}) and equilibrium constants (k_{+} , k_{-} and K) for CO_2 on Fe_3/Cu(100) surface at temperature from 250 to 1000 K. (Energy: kJ/mol Temperature: K k: s^{-1})

Temperature	ΔE_a^{+}	ΔE_a^{-}	k_{+}	k_{-}	$K(k_{+}/k_{-})$
250	56.98	96.44	7.64×10^{1}	1.11×10^{-7}	6.86×10^{8}
300	56.71	96.52	8.22×10^{3}	2.46×10^{-4}	3.34×10^{7}
350	56.43	96.66	2.33×10^{5}	5.91×10^{-2}	3.93×10^{6}
400	56.16	96.84	2.85×10^{6}	3.56×10^{0}	8.00×10^{5}
450	55.90	97.07	1.99×10^{7}	8.51×10^{1}	2.34×10^{5}
500	55.66	97.33	9.40×10^{7}	1.07×10^{3}	8.80×10^{4}
550	55.44	97.64	3.33×10^{8}	8.40×10^{3}	3.97×10^{4}
600	55.24	97.98	9.53×10^{8}	4.65×10^{4}	2.05×10^{4}
650	55.07	98.35	2.31×10^{9}	1.97×10^{5}	1.17×10^{4}
700	54.91	98.74	4.90×10^{9}	6.74×10^{5}	7.27×10^{3}
750	54.78	99.17	9.40×10^{9}	1.95×10^{6}	4.81×10^{3}
800	54.67	99.62	1.65×10^{10}	4.93×10^{6}	3.36×10^{3}
850	54.58	100.09	2.72×10^{10}	1.11×10^{7}	2.44×10^{3}
900	54.51	100.59	4.21×10^{10}	2.29×10^{7}	1.84×10^{3}
950	54.47	101.11	6.21×10^{10}	4.34×10^{7}	1.43×10^{3}
1000	54.44	101.65	8.80×10^{10}	7.72×10^{7}	1.14×10^{3}

Table S14 The forward and inverse corrected activatin energy barrier (ΔE_a^+ and ΔE_a^-) and equilibrium constants (k_+ , k_- and K) for CO_2 on Fe_4/Cu(100) surface at temperature from 250 to 1000 K. (Energy: kJ/mol Temperature: K k: s^{-1})

Temperature	ΔE_a^+	ΔE_a^-	k_+	k_-	$K(k_+/k_-)$
250	26.02	99.44	3.61×10^{8}	2.25×10^{-9}	1.60×10^{17}
300	25.46	100.54	3.63×10^{9}	4.21×10^{-6}	8.62×10^{14}
350	24.92	101.70	1.88×10^{10}	8.98×10^{-4}	2.10×10^{13}
400	24.39	102.90	6.45×10^{10}	4.93×10^{-2}	1.31×10^{12}
450	23.88	104.15	1.67×10^{11}	1.10×10^{0}	1.52×10^{11}
500	23.39	105.45	3.55×10^{11}	1.30×10^{1}	2.73×10^{10}
550	22.93	106.78	6.54×10^{11}	9.76×10^{1}	6.70×10^{9}
600	22.50	108.14	1.08×10^{12}	5.20×10^{2}	2.09×10^{9}
650	22.09	109.54	1.65×10^{12}	2.13×10^{3}	7.77×10^{8}
700	21.71	110.96	2.36×10^{12}	7.09×10^{3}	3.33×10^{8}
750	21.36	112.41	3.21×10^{12}	2.00×10^{4}	1.60×10^{8}
800	21.03	113.89	4.17×10^{12}	4.95×10^{4}	8.44×10^{7}
850	20.73	115.39	5.25×10^{12}	1.10×10^{5}	4.79×10^{7}
900	20.44	116.91	6.41×10^{12}	2.21×10^{5}	2.90×10^{7}
950	20.18	118.46	7.65×10^{12}	4.14×10^{5}	1.85×10^{7}
1000	19.94	120.02	8.95×10^{12}	7.26×10^{5}	1.23×10^{7}

Table S15 The forward and inverse corrected activatin energy barrier (ΔE_a^+ and ΔE_a^-) and equilibrium constants (k_+ , k_- and K) for CO_2 on Fe_5/Cu(100) surface at temperature from 250 to 1000 K. (Energy: kJ/mol Temperature: K k: s^{-1})

Temperature	ΔE_a^+	ΔE_a^-	k_+	k_-	$K(k_+/k_-)$
250	28.57	99.86	1.07×10^{8}	1.66×10^{-9}	6.48×10^{16}
300	28.01	100.97	1.33×10^{9}	3.20×10^{-6}	4.17×10^{14}
350	27.45	102.14	8.03×10^{9}	6.95×10^{-4}	1.16×10^{13}
400	26.91	103.36	3.07×10^{10}	3.86×10^{-2}	7.95×10^{11}
450	26.39	104.64	8.67×10^{10}	8.68×10^{-1}	9.99×10^{10}
500	25.89	105.96	1.98×10^{11}	1.04×10^{1}	1.91×10^{10}
550	25.43	107.32	3.86×10^{11}	7.81×10^{1}	4.94×10^{9}
600	24.99	108.71	6.69×10^{11}	4.17×10^{2}	1.61×10^{9}
650	24.58	110.14	1.06×10^{12}	1.71×10^{3}	6.21×10^{8}
700	24.19	111.60	1.57×10^{12}	5.71×10^{3}	2.75×10^{8}
750	23.83	113.09	2.20×10^{12}	1.62×10^{4}	1.36×10^{8}
800	23.49	114.60	2.93×10^{12}	4.00×10^{4}	7.33×10^{7}
850	23.18	116.14	3.77×10^{12}	8.86×10^{4}	4.26×10^{7}
900	22.89	117.70	4.70×10^{12}	1.79×10^{5}	2.62×10^{7}
950	22.62	119.28	5.72×10^{12}	3.36×10^{5}	1.70×10^{7}
1000	22.38	120.88	6.79×10^{12}	5.89×10^{5}	1.15×10^{7}

Table S16 The forward and inverse corrected activatin energy barrier (ΔE_a^+ and ΔE_a^-) and equilibrium constants (k_+ , k_- and K) for CO_2 on Fe_6/Cu(100) surface at temperature from 250 to 1000 K. (Energy: kJ/mol Temperature: K k: s^{-1})

Temperature	ΔE_a^+	ΔE_a^-	k_+	k_-	$K(k_+/k_-)$
250	32.01	112.17	3.16×10^{7}	4.45×10^{-12}	7.12×10^{18}
300	31.26	113.31	5.56×10^{8}	2.26×10^{-8}	2.46×10^{16}
350	30.52	114.51	4.30×10^{9}	9.87×10^{-6}	4.35×10^{14}
400	29.80	115.77	1.98×10^{10}	9.27×10^{-4}	2.14×10^{13}
450	29.10	117.06	6.46×10^{10}	3.13×10^{-2}	2.06×10^{12}
500	28.42	118.40	1.65×10^{11}	5.19×10^{-1}	3.19×10^{11}
550	27.78	119.78	3.55×10^{11}	5.12×10^{0}	6.94×10^{10}
600	27.16	121.19	6.67×10^{11}	3.42×10^{1}	1.95×10^{10}
650	26.56	122.63	1.13×10^{12}	1.70×10^{2}	6.66×10^{9}
700	26.00	124.10	1.77×10^{12}	6.67×10^{2}	2.66×10^{9}
750	25.46	125.60	2.60×10^{12}	2.17×10^{3}	1.20×10^{9}
800	24.94	127.12	3.63×10^{12}	6.09×10^{3}	5.96×10^{8}
850	24.45	128.66	4.85×10^{12}	1.51×10^{4}	3.22×10^{8}
900	23.98	130.23	6.26×10^{12}	3.36×10^{4}	1.86×10^{8}
950	23.53	131.82	7.84×10^{12}	6.86×10^{4}	1.14×10^{8}
1000	23.11	133.43	9.57×10^{12}	1.30×10^{5}	7.35×10^{7}

Table S17 The forward and inverse corrected activatin energy barrier (ΔE_a^+ and ΔE_a^-) and equilibrium constants (k_+ , k_- and K) for CO_2 on Fe_7/Cu(100) surface at temperature from 250 to 1000 K. (Energy: kJ/mol Temperature: K k: s^{-1})

Temperature	ΔE_a^+	ΔE_a^-	k_+	k_-	$K(k_+/k_-)$
250	29.38	155.77	3.76×10^{7}	9.26×10^{-21}	4.06×10^{27}
300	29.09	156.48	4.46×10^{8}	1.85×10^{-15}	2.41×10^{23}
350	28.81	157.25	2.60×10^{9}	1.11×10^{-11}	2.35×10^{20}
400	28.54	158.07	9.72×10^{9}	7.41×10^{-9}	1.31×10^{18}
450	28.29	158.94	2.69×10^{10}	1.15×10^{-6}	2.33×10^{16}
500	28.07	159.86	6.05×10^{10}	6.48×10^{-5}	9.34×10^{14}
550	27.87	160.82	1.17×10^{11}	1.73×10^{-3}	6.73×10^{13}
600	27.70	161.81	2.01×10^{11}	2.66×10^{-2}	7.53×10^{12}
650	27.56	162.83	3.16×10^{11}	2.67×10^{-1}	1.18×10^{12}
700	27.45	163.88	4.64×10^{11}	1.92×10^{0}	2.42×10^{11}
750	27.36	164.96	6.44×10^{11}	1.05×10^{1}	6.11×10^{10}
800	27.29	166.06	8.55×10^{11}	4.67×10^{1}	1.83×10^{10}
850	27.25	167.19	1.10×10^{12}	1.73×10^{2}	6.34×10^{9}
900	27.24	168.35	1.36×10^{12}	5.51×10^{2}	2.47×10^{9}
950	27.24	169.52	1.65×10^{12}	1.55×10^{3}	1.06×10^{9}
1000	27.27	170.72	1.95×10^{12}	3.93×10^{3}	4.96×10^{8}

Table S18 The forward and inverse corrected activatin energy barrier (ΔE_a^+ and ΔE_a^-) and equilibrium constants (k_+ , k_- and K) for CO_2 on Fe_8/Cu(100) surface at temperature from 250 to 1000 K. (Energy: kJ/mol Temperature: K k: s^{-1})

Temperature	ΔE_a^+	ΔE_a^-	k_+	k_-	$K(k_+/k_-)$
250	33.51	164.28	8.18×10^{6}	5.66×10^{-23}	1.44×10^{29}
300	33.04	165.43	1.45×10^{8}	1.88×10^{-17}	7.71×10^{24}
350	32.58	166.64	1.13×10^{9}	1.62×10^{-13}	6.98×10^{21}
400	32.13	167.91	5.22×10^{9}	1.42×10^{-10}	3.68×10^{19}
450	31.71	169.22	1.71×10^{10}	2.72×10^{-8}	6.28×10^{17}
500	31.32	170.58	4.38×10^{10}	1.81×10^{-6}	2.43×10^{16}
550	30.95	171.98	9.43×10^{10}	5.55×10^{-5}	1.70×10^{15}
600	30.60	173.41	1.78×10^{11}	9.57×10^{-4}	1.85×10^{14}
650	30.29	174.87	3.02×10^{11}	1.06×10^{-2}	2.85×10^{13}
700	29.99	176.36	4.73×10^{11}	8.27×10^{-2}	5.72×10^{12}
750	29.73	177.88	6.97×10^{11}	4.89×10^{-1}	1.42×10^{12}
800	29.49	179.42	9.73×10^{11}	2.31×10^{0}	4.22×10^{11}
850	29.27	180.98	1.30×10^{12}	9.04×10^{0}	1.44×10^{11}
900	29.07	182.56	1.68×10^{12}	3.03×10^{1}	5.55×10^{10}
950	28.89	184.17	2.11×10^{12}	8.94×10^{1}	2.36×10^{10}
1000	28.74	185.79	2.58×10^{12}	2.36×10^{2}	1.10×10^{10}

Table S19 The forward and inverse corrected activatin energy barrier (ΔE_a^+ and ΔE_a^-) and equilibrium constants (k_+ , k_- and K) for CO_2 on Fe_9/Cu(100) surface at temperature from 250 to 1000 K. (Energy: kJ/mol Temperature: K k: s^{-1})

Temperature	ΔE_a^+	ΔE_a^-	k_+	k_-	$K(k_+/k_-)$
250	26.44	159.14	6.28×10^{8}	8.53×10^{-21}	7.36×10^{28}
300	25.57	159.21	7.41×10^{9}	2.89×10^{-15}	2.57×10^{24}
350	24.71	159.35	4.30×10^{10}	2.51×10^{-11}	1.71×10^{21}
400	23.87	159.55	1.60×10^{11}	2.22×10^{-8}	7.20×10^{18}
450	23.05	159.79	4.42×10^{11}	4.30×10^{-6}	1.03×10^{17}
500	22.26	160.08	9.89×10^{11}	2.87×10^{-4}	3.45×10^{15}
550	21.50	160.41	1.90×10^{12}	8.85×10^{-3}	2.15×10^{14}
600	20.76	160.78	3.26×10^{12}	1.53×10^{-1}	2.13×10^{13}
650	20.05	161.17	5.13×10^{12}	1.70×10^{0}	3.02×10^{12}
700	19.37	161.60	7.52×10^{12}	1.33×10^{1}	5.66×10^{11}
750	18.71	162.05	1.04×10^{13}	7.87×10^{1}	1.33×10^{11}
800	18.07	162.52	1.38×10^{13}	3.72×10^{2}	3.72×10^{10}
850	17.46	163.02	1.77×10^{13}	1.46×10^{3}	1.21×10^{10}
900	16.87	163.54	2.20×10^{13}	4.90×10^{3}	4.49×10^{9}
950	16.30	164.09	2.66×10^{13}	1.44×10^{4}	1.84×10^{9}
1000	15.76	164.65	3.15×10^{13}	3.81×10^{4}	8.26×10^{8}
